# Associations of the exact-overlap Farm-to-Diet Coupling Index and production diversity with market dependence among smallholder farming households in Los Ríos, Ecuador: a cross-sectional study

**DOI:** 10.3389/fnut.2026.1923220

**Published:** 2026-09-08

**Authors:** Raynier Zambrano-Villacres, Jaen Cagua-Ordóñez, Daniel Jossepp Contreras-Moscol, Nelly Andrade Mejia, David Gortaire-Díaz, Daniel Simancas-Racines

**Affiliations:** 1 Facultad de Ciencias de la Salud y Desarrollo Humano, Universidad ECOTEC, Samborondón, Ecuador; 2 Escuela de Posgrado, Doctorado en Ciencias de la Salud, Instituto Universitario Italiano de Rosario (IUNIR), Rosario, Argentina; 3 Facultad de Ciencias de la Salud y Bienestar Humano, Universidad Tecnológica Indoamérica, Ambato, Ecuador; 4 Facultad de Ciencias Económicas y Empresariales, Universidad ECOTEC, Samborondón, Ecuador; 5 Facultad de Ciencias de la Salud, Universidad Espíritu Santo, Samborondón, Ecuador; 6 Dirección de Posgrados, Universidad ECOTEC, Samborondón, Ecuador

**Keywords:** farm-to-diet coupling, smallholder agriculture, food security, market dependence, production diversity, sustainable food systems

## Abstract

**Background:**

Smallholder farming households may produce food while remaining dependent on markets for household consumption. We examined whether household production diversity and exact production–self-consumption overlap were associated with market dependence, food insecurity, market-dependent vulnerability, and food-system buffer resilience among smallholder farming households in Los Ríos, Ecuador.

**Methods:**

We conducted a cross-sectional study of 133 households. Ten primary dietary food groups were classified as produced, self-consumed from own production, or purchased. The exact-overlap Farm-to-Diet Coupling Index (FDCI) represented the proportion of food groups produced that were also self-consumed. Modified Poisson models with HC3 robust standard errors were used to estimate adjusted prevalence ratios (aPRs).

**Results:**

Median household production diversity was 3 food groups (IQR: 1–4), the Self-consumed Food Group Diversity Proxy was 2 groups (IQR: 1–2), the FDCI was 0.50 (IQR 0.40–1.00), and the Market Food Dependency Index was 0.67 (IQR: 0.50–0.86). Production diversity was positively correlated with self-consumed food-group diversity (Spearman’s ρ = 0.636; *p* < 0.001). Vegetables, fish, and red meat showed the largest purchase–production differences. Each additional food group produced was associated with a lower prevalence of high market dependence (aPR 0.708; 95% CI 0.603–0.832), as was each 0.10-unit increase in the FDCI (aPR 0.933; 95% CI 0.874–0.996). The FDCI estimate was less precise in several sensitivity analyses and was not independently associated with food insecurity, market-dependent vulnerability, or buffer resilience.

**Conclusion:**

Production diversity and exact farm-to-diet overlap captured distinct dimensions of household food acquisition. Both were associated with lower market dependence, but exact overlap was not a consistent indicator of broader food insecurity, vulnerability, or resilience. These findings support assessing production, self-consumption, and purchase as distinct pathways rather than assuming that production diversification alone improves household food security. This distinction may help nutrition-sensitive agricultural programs monitor production, household retention, and market acquisition as separate but interconnected pathways.

## Introduction

Food security is shaped not only by agricultural supply but also by households’ capacity to obtain, consume, and sustain access to diverse and nutritious foods. In rural settings, this capacity depends on the interaction of agricultural production, market access, food purchases, income, financial resources, and exposure to economic or environmental shocks (1–3). Foods may be produced, sold, exchanged, stored, purchased, shared, or retained for household consumption through overlapping pathways. Farming households may therefore contribute to agricultural output while remaining dependent on markets or vulnerable to food insecurity when their production does not correspond to household food use or when economic buffers are limited (4, 5).

Smallholder households are central to this discussion because they participate in food systems as both producers and consumers (6). Although small and family farms account for a substantial share of agricultural holdings and contribute meaningfully to food production, agricultural participation does not necessarily ensure adequate household food access (7, 8). Food security also depends on how production is allocated between sale and household consumption, whether markets provide affordable foods that are not locally produced, and whether household resources are sufficient to maintain food acquisition (9).

Research has frequently examined whether greater farm production diversity is associated with improved dietary diversity. Households producing a wider range of food groups may have more opportunities to consume foods from their own farms (2, 10). However, the magnitude and consistency of this relationship vary across settings. Market access, commercialization, income, assets, seasonality, and local infrastructure may influence household diets alongside, and sometimes more strongly than, production diversity (3, 11, 12). Production and markets should therefore be considered complementary rather than competing pathways, because markets can provide foods that are seasonally unavailable, unsuitable for local production, or absent from the household production portfolio (4).

Production-diversity counts nevertheless provide limited information about how agricultural production is connected to household food use. Counting the number of food groups produced does not establish whether those same groups are retained and consumed by the household (13). Households may sell or exchange part of their production while continuing to purchase vegetables, animal-source foods, tubers, or other food groups. Conversely, they may report self-consuming foods that are not documented in the production inventory. Separate production and self-consumption counts may therefore conceal whether the same food groups are present in both sets. Measuring exact food-group overlap can complement conventional production-diversity indicators by estimating the proportion of the household production portfolio that is also confirmed as self-consumed (12).

Market dependence adds another dimension to this relationship. Food purchases may improve access to diverse foods when markets are affordable, accessible, and reliable, while market participation may generate income for foods that households do not produce (4, 14). Market reliance is therefore not inherently adverse. It may become a source of vulnerability when purchased foods account for a large share of household food acquisition and are combined with unstable income, limited savings, seasonal production gaps, transport barriers, or food-price volatility (5, 8). Productive, financial, and institutional resources may help households absorb these pressures, but they do not replace the need to examine production, self-consumption, and purchase as distinct and interconnected pathways (15–18).

This issue is particularly relevant in Los Ríos, one of Ecuador’s major agricultural provinces. Official statistics describe a productive structure that includes rice, banana, cacao, hard yellow maize, plantain, and livestock products, reflecting the coexistence of food crops, commercial agriculture, and animal production (19, 20). However, the agricultural importance of the province does not necessarily imply that farming households obtain most of their food from their own production. Evidence from rural Ecuador indicates that production diversity, own production, market foods, and other acquisition pathways make different contributions to household diets (21, 22). The extent to which the food groups produced by households are also retained for self-consumption, and how this overlap relates to market dependence, remains insufficiently characterized in Los Ríos.

To address this gap, we examined household production, self-consumption from own production, and market purchase across comparable food groups among smallholder farming households in Los Ríos. We operationalized farm-to-diet coupling as the proportion of food groups produced by a household that were also confirmed as self-consumed by that household. The resulting exact-overlap Farm-to-Diet Coupling Index was conceived as a complementary household-level measure rather than as a replacement for production diversity or a validated dietary diversity indicator. We also quantified food-group-specific differences between purchase and production prevalence to identify food groups that were predominantly market-sourced or production-dominant.

The primary objective was to examine whether household production diversity and exact farm-to-diet coupling were associated with market food dependence. Secondary objectives were to evaluate their associations with food insecurity, market-dependent food vulnerability, and food-system buffer resilience, and to assess the consistency of the FDCI findings across alternative definitions and model specifications. We hypothesized that greater production diversity would be associated with a broader range of food groups self-consumed from own production and that stronger exact farm-to-diet coupling would be associated with lower market dependence.

## Materials and methods

### Study design and setting

We conducted a cross-sectional household-level study among smallholder farming households in Los Ríos, Ecuador. The household was selected as the unit of analysis because the study examined the relationship between household food production, self-consumption from own production, market food acquisition, food insecurity, and food-system buffering capacity. The objective was not to estimate agricultural production at the provincial level, but to determine whether household production diversity was reflected in the food groups retained for household consumption and whether these production–consumption patterns were associated with reliance on purchased foods.

Los Ríos is one of Ecuador’s main agricultural provinces and combines food-crop production, livestock farming, commercial agriculture, and market-oriented smallholder production. This context allowed us to examine whether households actively involved in agriculture nevertheless depended on markets to obtain food groups for their own sustenance.

Data were collected through a structured household survey conducted in April 2025. The study was not designed to produce representative canton-level prevalence estimates. Canton-specific results were therefore restricted to exploratory descriptions of heterogeneity within the analytical sample.

### Study population, recruitment, and analytical sample

Eligible participants were smallholder farming households located in Los Ríos that reported agricultural or livestock production during the survey reference period. Respondents were adult household members familiar with the household’s productive activities, food acquisition, self-consumption practices, institutional support, and financial conditions.

Households were recruited during community-based field activities conducted in April 2025 with support from local agricultural organizations, community representatives, and territorial contacts. All households identified through these recruitment routes were screened using the same eligibility criteria and were invited to participate when an eligible adult respondent was available. Recruitment continued throughout the predefined data-collection period. Because the procedure was non-probabilistic, the study was designed to examine household-level associations and not to estimate population-representative prevalences for Los Ríos or its cantons.

The final dataset included 133 eligible households that completed the survey and had sufficient information for construction of the primary indicators. Descriptive analyses used all available observations, whereas regression models used complete cases according to the variables included in each model, resulting in analytical samples ranging from 122 to 124 households.

No formal *a priori* sample-size calculation was conducted because the study analyzed the complete set of eligible households recruited during the predefined fieldwork period. Model complexity was consequently restricted according to the available outcome events. The core models contained between 8.9 and 13.2 events per estimated parameter, while broader adjustment specifications were treated as sensitivity analyses. Model stability was evaluated through bootstrap resampling, penalized regression, cluster-robust inference, multicollinearity assessment, and influence diagnostics.

### Survey measures and recall framework

The survey collected information on household characteristics, agricultural and livestock production, self-consumed foods from own production, purchased foods, institutional support, financial resources, agroecological practices, and food insecurity-related conditions.

Agricultural and livestock self-consumption were assessed using the open-ended questions, “¿Qué productos agrícolas provenientes de su producción consumen en su hogar?” and “¿Qué productos pecuarios provenientes de su producción consumen en su hogar?” Food purchase was assessed separately using the corresponding questions on agricultural and livestock products purchased for daily sustenance. Respondents could report multiple products, which were subsequently separated, standardized, and classified into comparable food groups.

The questionnaire did not specify a standardized dietary recall period. Therefore, self-consumption was interpreted as a household-level food-group proxy rather than as a measure of individual dietary intake, consumption frequency, quantities consumed, nutrient adequacy, or validated dietary diversity. The original questions, response formats, recall framework, and analytical interpretations are reported in Supplementary Table 2.

### Data cleaning and harmonization

The original survey responses were preserved, and the analytical food-group variables were reconstructed from the coded and free-text responses using explicit and reproducible classification rules. Text entries were standardized for capitalization, accents, punctuation, spelling variants, and conjunctions. Responses containing multiple products were separated so that each item could be classified independently.

Classification rules were applied hierarchically to reduce false matches. Papaya, for example, was identified before potato-related terms to prevent its erroneous classification as a tuber. Foods of animal origin were assigned to species-specific groups whenever the source animal was reported. Generic references to meat without an identifiable animal source were retained as “nonspecific meat” and were not assigned to red meat, poultry, or pork.

Food groups reported as self-consumed but not recorded in the corresponding production inventory were retained as reported. However, these responses were not assumed to represent confirmed household production and did not contribute to the numerator of the exact-overlap Farm-to-Diet Coupling Index. Residual inconsistencies were documented through household- and food-group-level audits.

Data-quality checks examined duplicated household identifiers, missing observations, values outside the theoretical ranges of the indices, unexpected categories, inconsistencies between production and self-consumption, and implausible numeric values. Unusual observations were not automatically deleted unless an underlying data error or eligibility violation could be demonstrated. One extreme farm-size value was retained in the primary analysis and evaluated through exclusion, winsorization, and logarithmic-transformation sensitivity analyses.

### Food-group classification

Food products were classified into ten primary dietary food groups: cereals, grains, and legumes; tubers and roots; fruits; vegetables; red meat; white meat or poultry; pork; fish; dairy products; and eggs. This common classification was applied to production, self-consumption, and purchase records. Pork was retained as a separate food group because the original questionnaire and coding structure distinguished pork from other mammalian meats. This classification also preserved the food-group specificity of the original household responses. To evaluate whether this decision influenced the findings, the FDCI and the primary high-market-dependence model were recalculated after combining pork with red meat in a sensitivity analysis.

Commercial crops were retained as a separate production category for auditing and descriptive purposes but were excluded from the primary diversity and coupling indicators because they did not constitute a comparable dietary food group. Nonspecific meat responses were similarly retained for auditing but excluded from species-specific food groups.

For each primary food group, three household-level binary indicators were generated to identify whether the household produced the group, consumed it from its own production, or purchased it. These pathways were not mutually exclusive. A household could produce and purchase foods within the same group or could report both self-consumption from own production and market purchase.

### Household Production Diversity Score

The Household Production Diversity Score represented the number of primary food groups produced by household *i*:


$$H\,P\,D\,S_{i}=\overset{10}{\underset{g=1}{\sum }}P_{i\,g},$$

where *P*_*ig*_ = 1when household *i* reported producing food group *g*, and *P*_*ig*_ = 0 otherwise. Higher values indicated broader household food-production diversity.

### Self-consumed Food Group Diversity Proxy

The Self-consumed Food Group Diversity Proxy was calculated as:


$$S\,C\,F\,G\,D_{i}=\overset{10}{\underset{g=1}{\sum }}S_{i\,g},$$

where *S*_*ig*_ = 1 when household *i* reported consuming food group *g* from its own agricultural or livestock production.

The term “proxy” was used because the survey did not apply a validated dietary diversity instrument or a standardized dietary recall period. The measure therefore represents the diversity of food groups reportedly obtained from household production rather than total household dietary diversity.

### Exact-overlap Farm-to-Diet Coupling Index

The primary Farm-to-Diet Coupling Index quantified the proportion of a household’s production portfolio that was confirmed as reaching household self-consumption:


$$F\,D\,C\,I_{i}=\frac{\overset{10}{\underset{g=1}{\sum }}P_{i\,g}\,S_{i\,g}}{\overset{10}{\underset{g=1}{\sum }}P_{i\,g}},H\,P\,D\,S_{i}>0.$$

The numerator counted food groups that were both produced and self-consumed by the same household, while the denominator represented the total number of food groups produced. The FDCI ranged from 0 to 1, with higher values indicating that a larger proportion of the household production portfolio was retained for household consumption.

Households with no reported production among the primary dietary food groups had an undefined denominator and were treated as missing for the FDCI. Unlike the original self-consumed-to-produced group count ratio, the exact-overlap definition did not require truncation and did not assume that a self-consumed group had been produced unless a matching production record was available. The original truncated ratio, Jaccard similarity coefficient, and Sørensen similarity coefficient were retained as alternative definitions in sensitivity analyses.

### Preliminary evaluation of the Farm-to-Diet coupling index

The FDCI underwent a preliminary assessment of its conceptual coherence, distributional properties, convergent behavior, and robustness rather than formal psychometric or predictive validation. Content validity was addressed by defining the numerator through exact household-level matching of food groups reported as both produced and self-consumed and by auditing discordant production and self-consumption records. We examined the theoretical and observed ranges, median and interquartile range, boundary values, and the frequency of households whose original self-consumed-to-produced group-count ratio exceeded 1. Convergent behavior was explored through its relationships with the Household Production Diversity Score, the Self-consumed Food Group Diversity Proxy, and the Market Food Dependency Index.

Robustness was assessed by comparing the exact-overlap FDCI with the legacy truncated ratio, the Jaccard similarity coefficient, and the Sørensen similarity coefficient. Additional analyses combined pork with red meat and restricted the denominator to households producing at least two or at least three food groups. Because no external criterion measure or independent validation sample was available, the FDCI was interpreted as a novel operational index requiring further validation rather than as a validated measure of household diet quality or food-system resilience.

### Market Food Dependency Index

The Market Food Dependency Index represented the proportion of food groups obtained through purchase among all food groups reported as acquired through household self-consumption or purchase:


$$M\,F\,D\,I_{i}=\frac{\overset{10}{\underset{g=1}{\sum }}M_{i\,g}}{\overset{10}{\underset{g=1}{\sum }}I\left(S_{i\,g}=1\mathrm{or}M_{i\,g}=1\right)},$$

where *M*_*ig*_ = 1 when household *i* reported purchasing food group *g*, and *I* (⋅)denotes the indicator function. The index ranged from 0 to 1, with higher values indicating that a larger proportion of the household’s reported food-group acquisition depended on market purchase.

### Purchase–production difference and confirmed overlap by food group

For each food group, the difference between purchase and production prevalence was calculated as:


$$PPD_{g}=\mathrm{Pr}\left(M_{g}=1\right)-\mathrm{Pr}\left(P_{g}=1\right),$$

and expressed in percentage points. Positive values indicated that purchase prevalence exceeded production prevalence, whereas negative values indicated that production prevalence exceeded purchase prevalence. This was a descriptive prevalence difference and was not treated as a validated composite index.

Confirmed production–self-consumption overlap among producers was calculated as:


$$O\,v\,e\,r\,l\,a\,p_{g}=\frac{n\left(P_{g}=1\mathrm{and}S_{g}=1\right)}{n\left(P_{g}=1\right)}\times 100.$$

This measure represents the percentage of households producing a given group that also reported consuming that same group from their own production.

### Food insecurity, agroecological transition, and financial resilience

The food insecurity index was constructed from five survey items describing household conditions related to insufficient food availability or economic access to food. The index was scaled from 0 to 1, with higher values indicating greater food insecurity. Its internal consistency was evaluated using raw and standardized Cronbach’s alpha and the average inter-item correlation.

The Agroecological Transition Index was calculated as the equal-weight mean of nine binary components representing changes in cultivation practices, good agricultural practices, organic fertilization, environmentally appropriate waste disposal, reduced chemical use, elimination of crop-residue burning, soil conservation, use of agroecological inputs or practices, and training in good agricultural practices. Higher values indicated greater agroecological transition. The Financial Resilience Index summarized household financial resources and buffering capacity, with higher values indicating stronger financial resilience.

The agroecological transition and financial resilience indices were conceptualized as formative measures because their constituent variables represented complementary practices or resources rather than interchangeable manifestations of a single latent construct. Cronbach’s alpha estimates for these indices were therefore treated as descriptive and were not used to remove or retain individual components.

### Household Market-Dependent Food Vulnerability Index

The Household Market-Dependent Food Vulnerability Index was constructed as an equal-weight formative composite of market dependence, food insecurity, and limited financial resilience:


$$H\,M\,D\,F\,V\,I_{i}=\frac{M\,F\,D\,I_{i}+F\,I_{i}+\left(1-F\,R\,I_{i}\right)}{3},$$

where *FI_i_* denotes the food insecurity index and *FRI_i_* denotes the Financial Resilience Index. All three components were required to calculate the primary index. Values ranged from 0 to 1, with higher values indicating greater vulnerability characterized by reliance on purchased food, food insecurity, and limited financial buffering.

Leave-one-component-out versions and alternative specifications assigning greater weight to either market dependence or financial resilience were examined to determine whether the results depended on equal weighting.

### Food system buffer resilience index

To avoid circularity when evaluating production diversity and farm-to-diet coupling, the primary resilience outcome did not contain HPDS, self-consumed food-group diversity, FDCI, or MFDI. The Food System Buffer Resilience Index was calculated as the equal-weight mean of food security, agroecological transition, and financial resilience:


$$B\,u\,f\,f\,e\,r\,R\,e\,s\,i\,l\,i\,e\,n\,c\,e_{i}=\frac{\left(1-F\,I_{i}\right)+A\,T\,I_{i}+F\,R\,I_{i}}{3},$$

where *ATI_i_* denotes the Agroecological Transition Index. The index ranged from 0 to 1 and was multiplied by 100 for descriptive presentation. Higher values indicated stronger household buffering capacity independent of the production–diet indicators evaluated as exposures. Leave-one-component-out versions were constructed to examine the dependence of the index on any single component.

### Outcome definitions

Four binary outcomes were evaluated in the primary regression analysis. High market dependence was defined as an MFDI of at least 0.75. This operational threshold was selected to identify households obtaining a large proportion of their reported food groups through purchase. Because the cut-off had not been externally validated, a threshold of 0.50 and continuous MFDI models were used as sensitivity analyses.

High food insecurity was defined as a food insecurity index at or above the sample median. High market-dependent food vulnerability was defined as an HMDFVI at or above the sample median. Low food-system buffer resilience was defined as a Buffer Resilience Index below the sample median. Median-based thresholds were used because validated population cut-offs were unavailable for these locally constructed indices. Continuous versions of the four outcomes were also analyzed to evaluate whether the conclusions depended on dichotomization.

### Covariates and model specification

Covariates were selected *a priori* according to their conceptual relationship with household productive capacity, food acquisition, resource availability, and buffering capacity. Parsimonious core models were prioritized because of the modest sample size and available number of events.

Respondent gender was modeled as woman versus man. Farm size was entered continuously in hectares, household size as number of members, and age as years. Educational attainment was modeled as an ordinal variable ranging from no schooling or incomplete primary education to higher education.

Four separate core models were fitted. The high-market-dependence model included the exact-overlap FDCI, HPDS, respondent gender, farm size, and household size. The high-food-insecurity model included the exact-overlap FDCI, MFDI, respondent gender, farm size, and household size. The high market-dependent food vulnerability model included the exact-overlap FDCI, HPDS, respondent gender, farm size, and household size. The low-buffer-resilience model included the exact-overlap FDCI, MFDI, respondent gender, age, farm size, household size, and educational attainment.

Farm-to-Diet Coupling Index and MFDI were rescaled so that their regression estimates represented a 0.10-unit increase. HPDS estimates represented one additional food group produced. Extended models added selected institutional, financial, or agroecological covariates according to the outcome being examined. Because these models contained fewer events per estimated parameter, they were interpreted as sensitivity analyses rather than as the primary specifications.

### Descriptive and correlation analyses

Categorical variables were summarized using frequencies and percentages. Continuous, bounded, ordinal, and count variables were summarized using medians and interquartile ranges. Food-group production, self-consumption, and purchase percentages were calculated using all 133 households as the denominator and were not expected to sum to 100% because the acquisition pathways could overlap.

Spearman rank correlations were used to examine associations among the primary food-system indicators because several variables were bounded, skewed, ordinal, or count-based. Composite indices were excluded from the primary correlation heatmap when their inclusion would have produced part–whole correlations with their constituent components. Correlations were interpreted as descriptive associations and not as evidence of causal relationships.

### Production diversity and self-consumed food-group diversity

The unadjusted relationship between HPDS and the Self-consumed Food Group Diversity Proxy was described using Spearman’s rank correlation. A count regression model was subsequently fitted with the number of self-consumed food groups as the outcome and HPDS as the principal exposure, adjusted for respondent gender, farm size, and household size.

Poisson and negative binomial specifications were compared using the Akaike Information Criterion and the Pearson dispersion ratio. The Poisson model was retained because there was no evidence of overdispersion and the negative binomial specification did not improve model fit. HC3 heteroskedasticity-consistent standard errors were used for statistical inference.

### Modified Poisson regression

Modified Poisson regression with a log link and HC3 heteroskedasticity-consistent standard errors was used to estimate adjusted prevalence ratios and 95% confidence intervals for the four binary outcomes:


$$\log\,\left\{E\,\left(Y_{i}|X_{i}\right)\right\}=\beta_{0}+\beta_{1}\,X_{1\,i}+\cdots +\beta_{k}\,X_{k\,i}.$$

Adjusted prevalence ratios were obtained as:


$$a\,P\,R_{j}=\exp\,\left(\beta_{j}\right).$$

Modified Poisson regression was selected because the outcomes were common and prevalence ratios provided a more direct interpretation than odds ratios. Two-sided *p*-values were reported, although interpretation considered the magnitude and direction of the associations, confidence-interval width, consistency across related models, and sensitivity analyses rather than statistical significance alone.

### Continuous-outcome analyses

Continuous versions of MFDI, food insecurity, HMDFVI, and the Buffer Resilience Index were evaluated using fractional-logit models with HC3 robust standard errors. These analyses were used to assess whether the results were sensitive to sample-based dichotomization.

Average marginal effects were calculated on the response scale for the FDCI and expressed as the expected absolute change in the corresponding continuous outcome associated with a 0.10-unit increase in farm-to-diet coupling.

### Model stability and diagnostics

For each binary model, we documented the number of complete observations, events, non-events, estimated parameters, and events per parameter. Multicollinearity was assessed using variance inflation factors. The functional form of continuous predictors was evaluated by comparing models containing linear terms with models using natural splines. Splines were not retained when they did not sufficiently improve the Akaike Information Criterion to justify the additional model complexity.

Pearson dispersion ratios were examined as diagnostics of model behavior. In modified Poisson models for binary outcomes, these ratios were not treated as strict distributional assumptions because statistical inference was based on robust standard errors.

Influence was evaluated using Cook’s distance, leverage, Pearson residuals, deviance residuals, and DFBETAs. Observations exceeding the conventional Cook’s distance threshold of 4/*n* were reviewed but were not deleted automatically. The high-market-dependence model was refitted after excluding each influential observation separately and after excluding all influential observations jointly. The complete-sample model remained the primary analysis because no underlying data error or eligibility violation was identified.

### Sensitivity analyses

Robustness was examined using CR2 small-sample-corrected cluster-robust standard errors by canton, nonparametric bootstrap resampling with 2,000 replications, conventional logistic regression, and Firth penalized logistic regression. Crude, core, and extended adjustment specifications were compared to assess changes in the magnitude and precision of the FDCI estimate after adjustment for production diversity, household characteristics, and selected institutional or agroecological covariates. Because only ten cantonal clusters were available, cluster-robust estimates were interpreted cautiously and were treated as sensitivity analyses rather than as the primary inferential results.

Additional analyses evaluated continuous versions of the outcomes and an alternative threshold of 0.50 for high market dependence. Influence sensitivity analyses excluded observations with Cook’s distance greater than 4/n, first individually and then jointly. The potential influence of the extreme farm-size observation was examined through exclusion, winsorization, and logarithmic transformation of farm size. The complete-sample models using the prespecified variable definitions remained the primary analyses unless an underlying data error or eligibility violation was identified.

Farm-to-Diet Coupling Index-specific sensitivity analyses–including comparison with the legacy truncated ratio, Jaccard similarity coefficient, and Sørensen similarity coefficient; reclassification combining pork with red meat; and restriction to households producing at least two or at least three food groups–were conducted as described under “Preliminary evaluation of the Farm-to-Diet Coupling Index.” Restricted-denominator models were considered exploratory and were not interpreted as evidence of effect modification. Leave-one-component-out and alternative-weighting specifications were also used to assess the stability of the formative market-dependent vulnerability and buffer-resilience indices. Canton-specific findings were limited to descriptive summaries and cluster-robust sensitivity analyses because the study was not designed to estimate representative canton-level effects.

As an additional sensitivity analysis, we residualized the exact-overlap FDCI with respect to household production diversity. In the complete-case sample used for the primary market-dependence model, the FDCI was regressed on the Household Production Diversity Score using ordinary least squares. The resulting residuals represented the component of farm-to-diet coupling above or below the value expected from production diversity. These residuals were rescaled so that estimates corresponded to a 0.10-unit increase and were entered into a modified Poisson model adjusted for respondent gender, farm size, and household size. A reparameterized model retaining HPDS was also fitted as an internal consistency check. The simultaneous-adjustment model containing the original FDCI and HPDS remained the primary specification.

### Exploratory subgroup analyses

Respondent-gender and educational-attainment summaries were generated to describe within-sample heterogeneity. Canton-level summaries were also produced descriptively. These analyses were not interpreted as representative subgroup or canton-level prevalence estimates because several strata contained small numbers of households.

Household clustering and mediation analyses included in earlier analytical versions were not retained in the final framework. The clustering solution did not demonstrate sufficient empirical stability for substantive interpretation, and the cross-sectional design could not establish the temporal ordering required for mediation analysis.

### Statistical software

Analyses were conducted using R version 4.5 in RStudio. Data cleaning, indicator construction, descriptive analysis, regression modeling, model diagnostics, sensitivity analyses, and figure production were implemented through reproducible scripts. The principal packages included tidyverse, gtsummary, ggplot2, sandwich, lmtest, broom, MASS, logistf, clubSandwich, boot, marginaleffects, psych, performance, openxlsx, flextable, and officer.

### Ethical considerations

The study involved a household survey and did not use patient information, medical records, or retrospective clinical data. Before participation, respondents received information about the study objectives, procedures, voluntary nature of participation, confidentiality protections, and their right to decline or withdraw without consequences. Verbal informed consent was obtained from every respondent before questionnaire administration and was documented by the interviewer in accordance with the procedure approved by the ethics committee.

Personal identifiers were removed from the analytical dataset, and access to the data was restricted to the research team. Ethical approval was granted by the Institutional Academic Review Committee of Universidad Tecnológica Indoamérica, Department of Postgraduate Studies in Health, approval number UTI-DPS-IRB-038-2024.

## Results

### Study population, data completeness, and household food-system profile

The study included 133 smallholder farming households from Los Ríos, Ecuador (Table 1). Most respondents were men (109/133; 82.0%), and the median age was 46 years (IQR: 37–58). Median household size was 5 members (IQR: 4–6), median farm size was 5 hectares (IQR: 3–10), and median time engaged in agricultural or livestock production was 14 years (IQR: 5–25). Livestock production was reported by 70 households (52.6%), whereas 37 (27.8%) had received technical assistance, 49 of 127 households with available data (38.6%) reported access to agricultural credit, 86 (64.7%) reported savings, and 34 of 130 (26.2%) belonged to an agricultural association.

**TABLE 1:** Sociodemographic, productive, institutional, and food-system characteristics of smallholder farming households in Los Ríos, Ecuador.

Characteristic	Overall, *N* = 133
Sociodemographic characteristics	
Respondent gender	
Man	109 (82.0%)
Woman	24 (18.0%)
Age, years	46.0 (37.0–58.0)
Educational attainment	
No schooling or incomplete primary	18 (13.5%)
Complete primary	36 (27.1%)
Incomplete secondary	21 (15.8%)
Complete secondary	31 (23.3%)
Higher education	27 (20.3%)
Household size, members	5.0 (4.0–6.0)
Productive and institutional characteristics	
Farm size, hectares	5.0 (3.0–10.0)
Years in agricultural or livestock production	14.0 (5.0–25.0)
Livestock production	70 (52.6%)
Technical assistance	37 (27.8%)
Agricultural credit	49/127 (38.6%)
Agricultural insurance	31 (23.3%)
Savings	86 (64.7%)
Association membership	34/130 (26.2%)
Household food-system indicators	
Household Production Diversity Score, food groups	3.00 (1.00–4.00)
Self-consumed Food Group Diversity Proxy, food groups	2.00 (1.00–2.00)
Farm-to-Diet Coupling Index, exact-overlap definition	0.50 (0.40–1.00)
Market Food Dependency Index	0.67 (0.50–0.86)
Household Market-Dependent Food Vulnerability Index	0.50 (0.39–0.63)
Food insecurity index	0.30 (0.00–0.60)
Food System Buffer Resilience Index, 0–100	52.22 (41.48–64.81)
Agroecological transition index	0.44 (0.25–0.56)
Financial Resilience Index	0.50 (0.33–0.60)

Values are presented as median (interquartile range) or *n* (%). Percentages were calculated using available data; denominators are shown when they differ from 133. The exact-overlap FDCI represents the proportion of food groups produced that were also self-consumed. Higher buffer-resilience scores indicate greater household buffering capacity. IQR, interquartile range.

The median Household Production Diversity Score (HPDS) was 3 food groups (IQR: 1–4), whereas the median Self-consumed Food Group Diversity Proxy was 2 groups (IQR: 1–2). The exact-overlap Farm-to-Diet Coupling Index (FDCI) had a median of 0.50 (IQR: 0.40–1.00), and the median Market Food Dependency Index (MFDI) was 0.67 (IQR: 0.50–0.86). The median Household Market-Dependent Food Vulnerability Index was 0.50 (IQR: 0.39–0.63), while the median Food System Buffer Resilience Index was 52.22 on a scale from 0 to 100 (IQR: 41.48–64.81). The food insecurity index had a median of 0.30 (IQR: 0.00–0.60).

Missingness was limited. Agricultural credit had the highest proportion of missing observations (4.5%), followed by the MFDI and its derived outcomes (3.0%). Missingness in the FDCI, food insecurity index, household size, and agroecological transition index was 1.5% for each variable. Consequently, the main regression models included between 122 and 124 households. Multiple imputation was not performed because no selected model variable exceeded 5% missingness. Missing-data frequencies are reported in Supplementary Table 12 and illustrated in Supplementary Figure 5, while complete-case flow counts are presented in Supplementary Table 8.

The five-item food insecurity scale had a Cronbach’s alpha of 0.770, a standardized alpha of 0.760, and an average inter-item correlation of 0.388. Reliability coefficients for the agroecological transition and financial resilience indices were treated as descriptive because these indices were specified as formative rather than reflective measures. Detailed missing-data and reliability diagnostics are presented in Supplementary Table 12.

### Preliminary evaluation and audit of the Farm-to-Diet coupling index

Two households reported no food-group production and therefore had no defined ratio-based FDCI. Among the 131 households producing at least one food group, the original self-consumed-to-produced group ratio had a median of 0.60 (IQR: 0.50–1.00) and ranged from 0 to 3. Six households, representing 4.6% of those with a calculable ratio, had values greater than 1. These values occurred when the number of self-consumed food groups exceeded the number of groups recorded in the household production inventory.

The primary exact-overlap FDCI counted only food groups that were both produced and self-consumed by the same household. Its median was 0.50 (IQR: 0.40–1.00), with an observed and theoretical range from 0 to 1. Eighteen households (13.5%) had at least one self-consumed food group without a corresponding production record. These unconfirmed matches were not included in the numerator of the exact-overlap FDCI.

The household-level audit, discordances by food group, and comparisons among the exact-overlap, legacy, Jaccard, and Sørensen definitions are presented in Supplementary Table 3–5 and Supplementary Figures 2–4.

### Production, self-consumption, and purchase patterns by food group

**FIGURE 1:**
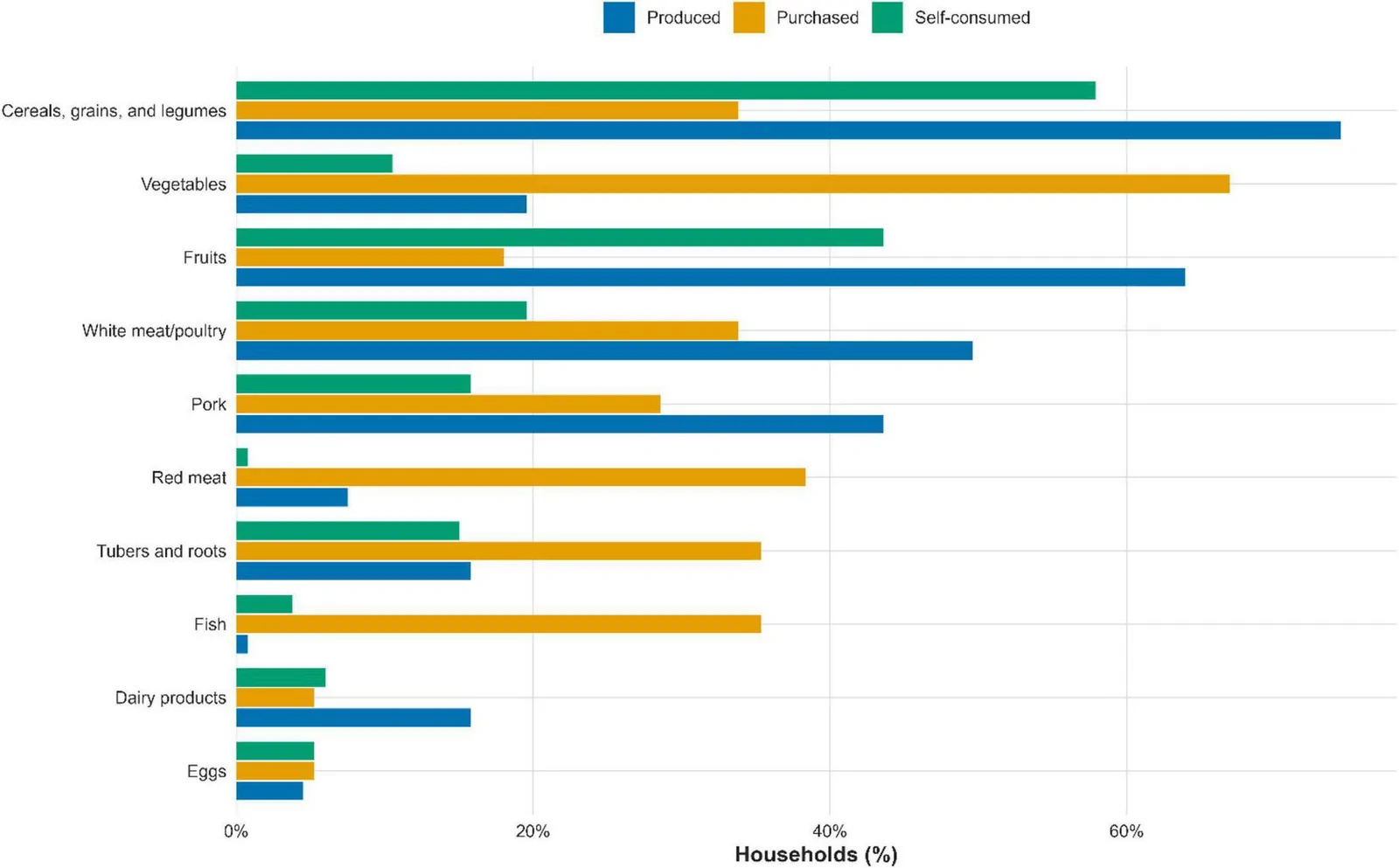
Production, self-consumption, and purchase by food group among smallholder farming households in Los Ríos, Ecuador. Bars show the percentage of all 133 households reporting production, purchase, or self-consumption from household production for each food group. These pathways are not mutually exclusive; therefore, the three percentages within a food group should not be interpreted as summing to 100%. Food groups are ordered according to the household production and food-acquisition patterns observed in the sample.

**FIGURE 2:**
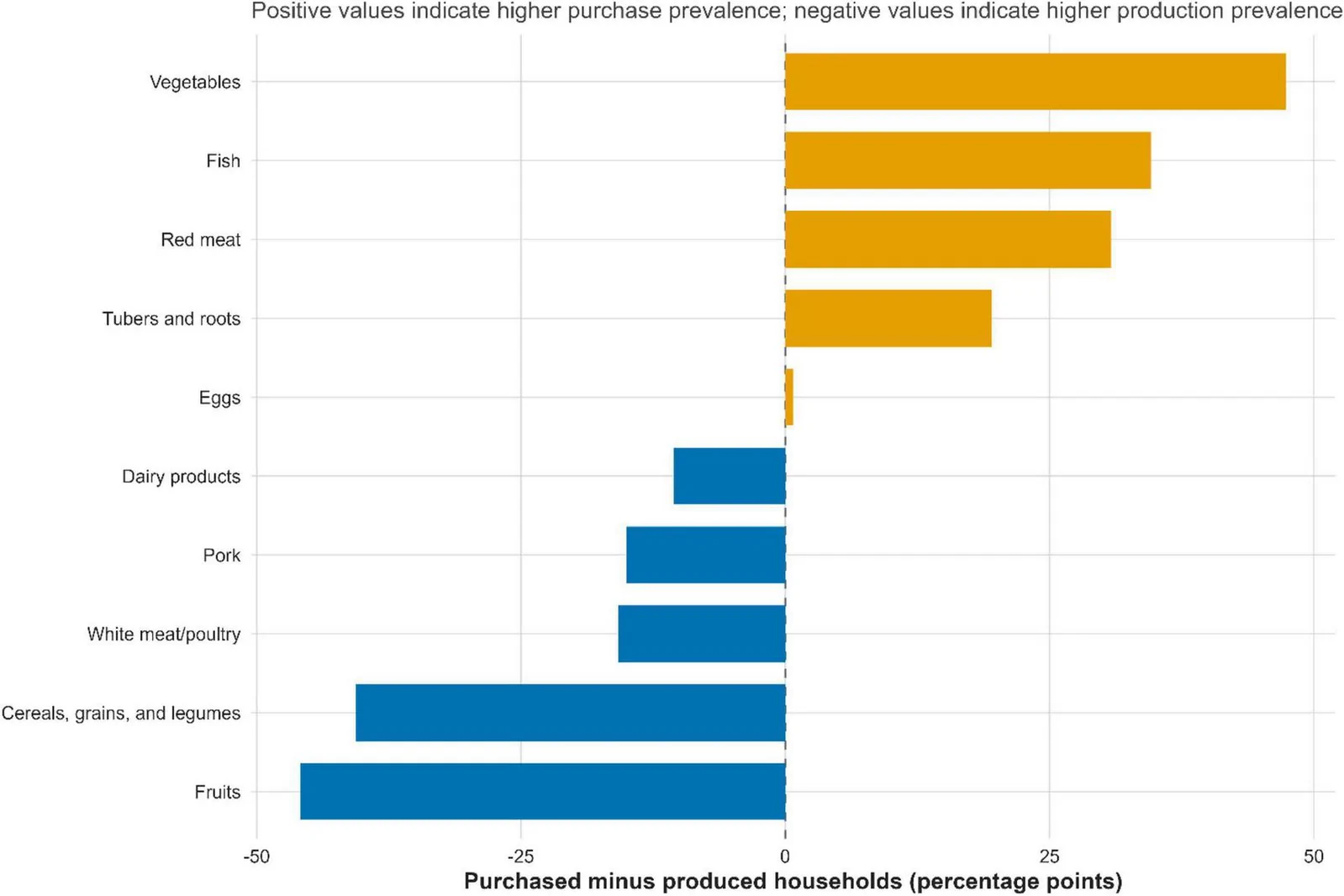
Difference between household purchase and production prevalence by food group. Bars represent the percentage of households purchasing each food group minus the percentage producing that group. Positive values indicate higher purchase prevalence and greater reliance on market acquisition, whereas negative values indicate higher household production prevalence. The dashed vertical line represents no difference between purchase and production. Values are descriptive differences in household prevalence and should not be interpreted as a composite index.

Production, self-consumption, and purchase varied substantially across food groups, and these pathways were not mutually exclusive. Cereals, grains, and legumes and fruits had the highest production prevalence and comparatively high confirmed production–self-consumption overlap. In contrast, vegetables, fish, and red meat showed the largest positive purchase–production differences, indicating that these groups were obtained predominantly through market purchase. Fruits and cereals, grains, and legumes showed the largest production-dominant differences. Detailed food-group estimates are presented in Table 2 and Figures 1, 2.

**TABLE 2:** Household production, self-consumption, purchase, and confirmed production–self-consumption overlap by food group.

Food group	Produced, *n* (%)	Self-consumed, *n* (%)	Purchased, *n* (%)	Confirmed production–self-consumption overlap, *n*	Self-consumption conversion among producers, %	Production–purchase gap, percentage points
Vegetables	26 (19.5)	14 (10.5)	89 (66.9)	12	46.2	47.4
Fish	1 (0.8)	5 (3.8)	47 (35.3)	1	100.0	34.6
Red meat	10 (7.5)	1 (0.8)	51 (38.3)	1	10.0	30.8
Tubers and roots	21 (15.8)	20 (15.0)	47 (35.3)	14	66.7	19.5
Eggs	6 (4.5)	7 (5.3)	7 (5.3)	6	100.0	0.8
Dairy products	21 (15.8)	8 (6.0)	7 (5.3)	7	33.3	−10.5
Pork	58 (43.6)	21 (15.8)	38 (28.6)	17	29.3	−15.0
White meat/poultry	66 (49.6)	26 (19.5)	45 (33.8)	23	34.8	−15.8
Cereals, grains, and legumes	99 (74.4)	77 (57.9)	45 (33.8)	75	75.8	−40.6
Fruits	85 (63.9)	58 (43.6)	24 (18.0)	54	63.5	−45.9

Values are *n* (%) unless otherwise indicated. Percentages were calculated using all 133 households. Overlap represents households that both produced and self-consumed the same food group. Conversion was calculated as overlap divided by the number of producers. The production–purchase gap equals purchased percentage minus produced percentage; positive values indicate greater reliance on purchases.

As shown in Figure 2, production prevalence exceeded purchase prevalence for fruits, cereals, grains and legumes, white meat or poultry, pork, and dairy products. The largest production-dominant differences were observed for fruits and cereals, grains and legumes.

Among producers, confirmed production–self-consumption overlap was generally highest for cereals, grains, and legumes, tubers and roots, and fruits; the apparent complete conversion for fish and eggs should be interpreted cautiously because very few households produced these groups (Table 2).

### Production diversity and self-consumed food-group diversity

**FIGURE 3:**
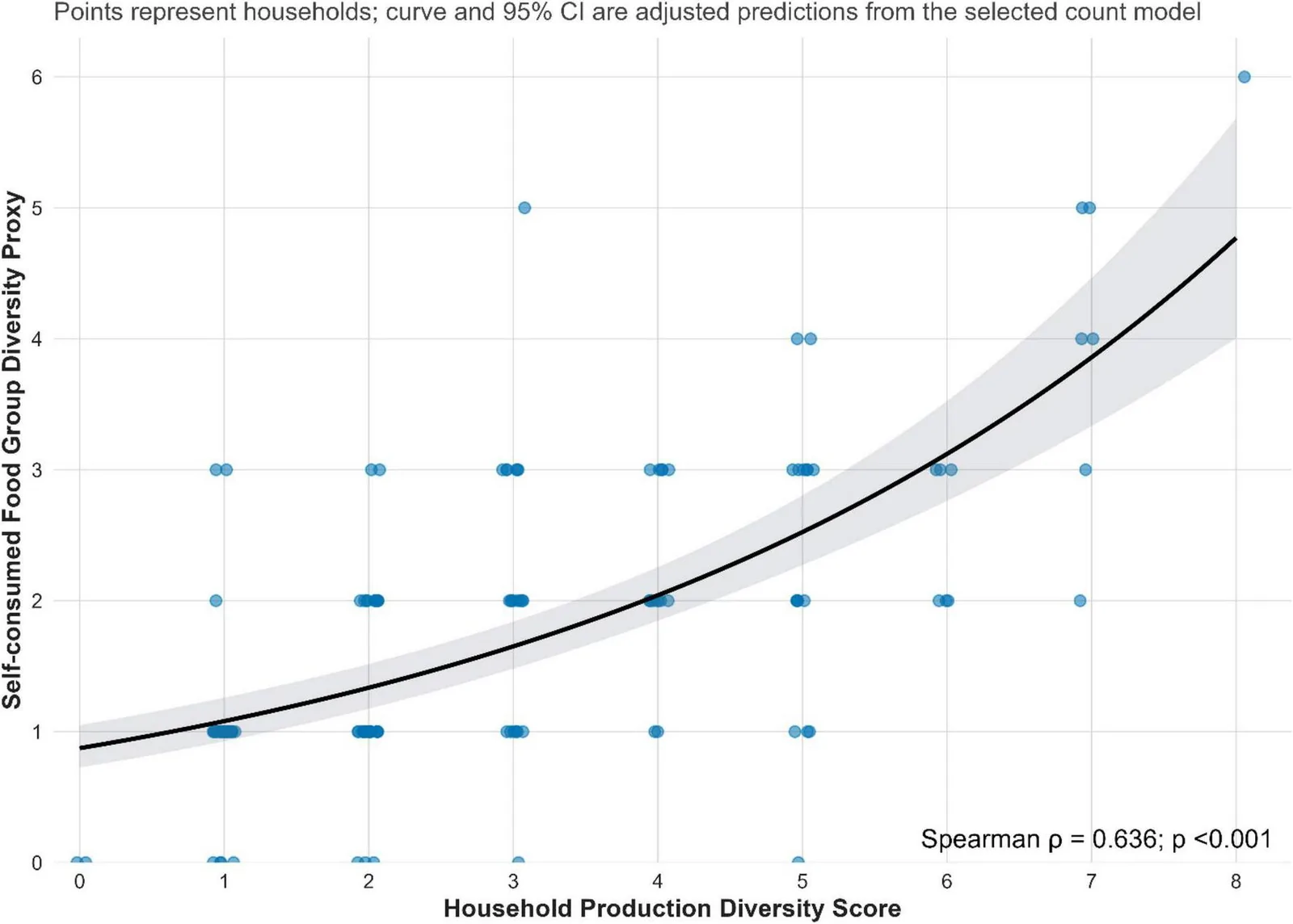
Association between household production diversity and self-consumed food-group diversity. Points represent individual households. The *x*-axis shows the Household Production Diversity Score, and the *y*-axis shows the Self-consumed Food Group Diversity Proxy. The solid curve and shaded band represent adjusted predictions and robust 95% confidence intervals from the selected Poisson count model, adjusted for respondent gender, farm size, and household size. Spearman’s rank correlation is displayed as a descriptive measure of the unadjusted association. The figure represents a cross-sectional relationship and should not be interpreted causally.

Household production diversity was positively correlated with self-consumed food-group diversity (Spearman’s ρ = 0.636; *p* < 0.001), as shown in Figure 3. The complete correlation matrix of the primary household food-system indicators is presented in Supplementary Table 1 and Supplementary Figure 1. In the adjusted count model, each additional food group produced was associated with a 23.6% higher expected number of self-consumed food groups (rate ratio: 1.236; 95% CI: 1.191–1.283; *p* < 0.001). Respondent gender and farm size were not independently associated with the outcome, whereas household size showed a small inverse association. Complete model coefficients and the comparison between the Poisson and negative binomial specifications are presented in Supplementary Table 7.

A Poisson specification was retained because it had a slightly lower Akaike Information Criterion than the negative binomial model (363.64 versus 365.64), and the Pearson dispersion ratio did not indicate overdispersion. Household size was weakly inversely associated with the expected number of self-consumed groups (rate ratio: 0.944; 95% CI: 0.891–0.999; *p* = 0.047), whereas gender and farm size were not independently associated with this outcome.

### Associations with market dependence

The primary high-market-dependence model included 124 households, of whom 49 (39.5%) met the operational MFDI threshold of 0.75. The model estimated five parameters excluding the intercept, corresponding to 9.8 events per parameter.

After adjustment for production diversity, respondent gender, farm size, and household size, each additional food group produced was associated with a 29.2% lower prevalence of high market dependence (adjusted PR: 0.708; 95% CI: 0.603–0.832; *p* < 0.001). Each 0.10-unit increase in the exact-overlap FDCI was associated with a 6.7% lower prevalence of high market dependence (adjusted PR: 0.933; 95% CI: 0.874–0.996; *p* = 0.037). Gender, farm size, and household size were not independently associated with this outcome.

The continuous MFDI analysis was consistent with the primary binary model. In the fractional-logit model, a 0.10-unit increase in the FDCI was associated with an average marginal decrease of 0.0215 in the MFDI, equivalent to approximately 2.15 percentage points (95% CI: −4.09 to −0.20 percentage points; *p* = 0.030). Production diversity was also inversely associated with continuous MFDI (coefficient: −0.196; 95% CI: −0.317 to −0.074; *p* = 0.002). Complete fractional-logit coefficients and average marginal effects are presented in Supplementary Table 6.

The FDCI estimate differed across progressive adjustment specifications. In the unadjusted model, the FDCI was not associated with high market dependence (PR: 0.999; 95% CI: 0.930–1.073; *p* = 0.976). The inverse association appeared after adjustment for production diversity and household covariates. In the extended model, which additionally included technical assistance, agricultural credit, and agroecological transition, the estimate remained inverse but was less precise (PR: 0.948; 95% CI: 0.881–1.021; *p* = 0.161).

### Food insecurity, market-dependent vulnerability, and buffer resilience

The high-food-insecurity model included 124 households and 66 events, corresponding to 13.2 events per parameter. Neither the exact-overlap FDCI (adjusted PR per 0.10-unit increase: 0.982; 95% CI: 0.930–1.038; *p* = 0.519) nor market dependence (adjusted PR per 0.10-unit increase: 1.040; 95% CI: 0.972–1.113; *p* = 0.255) was independently associated with high food insecurity. The continuous-outcome model produced concordant results, with no association between FDCI and food insecurity (average marginal effect: −0.0038; 95% CI: −0.0212 to 0.0137; *p* = 0.673). Market dependence had a positive but imprecise coefficient in the continuous model (*p* = 0.074). Complete continuous-outcome estimates are presented in Supplementary Table 6.

The high market-dependent vulnerability model included 124 households and 61 events, corresponding to 12.2 events per parameter. The adjusted PR was 0.953 per 0.10-unit increase in the FDCI (95% CI: 0.896–1.013; *p* = 0.123) and 0.968 per additional produced food group (95% CI: 0.865–1.084; *p* = 0.574). In the continuous model, the average marginal effect of a 0.10-unit FDCI increase was −0.0097 (95% CI: −0.0205 to 0.0010; *p* = 0.075; Supplementary Table 6). Component-removal and alternative-weighting analyses produced estimates that varied according to the construction of the vulnerability index (Supplementary Table 13).

The low buffer-resilience model included 122 households and 62 events, corresponding to 8.9 events per parameter. The FDCI was not associated with low buffer resilience (adjusted PR per 0.10-unit increase: 1.025; 95% CI: 0.965–1.090; *p* = 0.422). Market dependence had an adjusted PR of 1.059 per 0.10-unit increase (95% CI: 0.993–1.130; *p* = 0.082).

**FIGURE 4:**
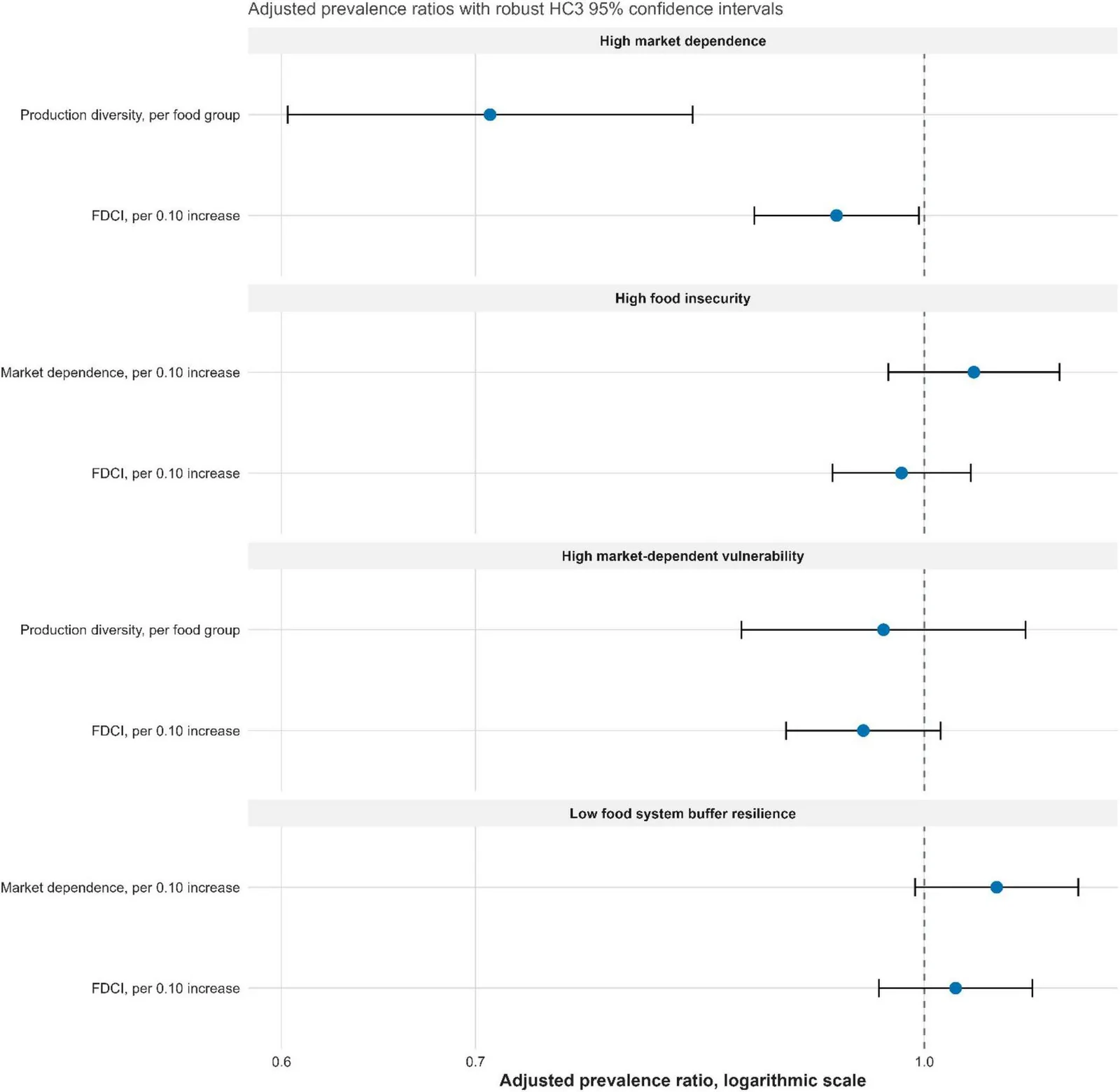
Core modified Poisson models for household food-system outcomes. The forest plot presents selected adjusted prevalence ratios and robust HC3 95% confidence intervals from the core modified Poisson regression models. FDCI and MFDI effects are reported per 0.10-unit increase, whereas production diversity is reported per additional food group. The dashed vertical line indicates a prevalence ratio of 1. Values below 1 indicate lower prevalence of the corresponding outcome, and values above 1 indicate higher prevalence. The *x*-axis is displayed on a logarithmic scale. Complete model results, including all adjustment variables, are presented in Table 3.

Women respondents had a higher prevalence of low buffer resilience than men respondents (adjusted PR: 1.755; 95% CI: 1.158–2.658; *p* = 0.008). Age, farm size, household size, and educational attainment were not independently associated with the binary outcome. In the continuous model, women respondents had a lower buffer-resilience score (coefficient: −0.380; 95% CI: −0.739 to −0.021; *p* = 0.038), whereas neither FDCI nor MFDI was associated with the continuous score (Supplementary Table 6). The core adjusted estimates for the four food-system outcomes are summarized in Figure 4 and Table 3.

**TABLE 3:** Core modified Poisson regression models for household food-system outcomes.

Outcome, events/*N*	Predictor	aPR (95% CI)	*P-*value
High market dependence, 49/124^a^	FDCI, per 0.10-unit increase	0.933 (0.874–0.996)	0.037
	HPDS, per additional food group	0.708 (0.603–0.832)	<0.001
	Respondent gender: woman vs. man	0.805 (0.430–1.505)	0.497
	Farm size, per hectare	1.001 (0.985–1.018)	0.879
	Household size, per member	0.950 (0.832–1.085)	0.452
High food insecurity, 66/124^b^	FDCI, per 0.10-unit increase	0.982 (0.930–1.038)	0.519
	MFDI, per 0.10-unit increase	1.040 (0.972–1.113)	0.255
	Respondent gender: woman vs. man	1.033 (0.679–1.570)	0.881
	Farm size, per hectare	0.987 (0.970–1.004)	0.132
	Household size, per member	1.066 (0.962–1.181)	0.223
High market-dependent food vulnerability, 61/124^c^	FDCI, per 0.10-unit increase	0.953 (0.896–1.013)	0.123
	HPDS, per additional food group	0.968 (0.865–1.084)	0.574
	Respondent gender: woman vs. man	1.314 (0.867–1.992)	0.198
	Farm size, per hectare	0.990 (0.975–1.006)	0.222
	Household size, per member	0.994 (0.881–1.122)	0.922
Low food-system buffer resilience, 62/122^d^	FDCI, per 0.10-unit increase	1.025 (0.965–1.090)	0.422
	MFDI, per 0.10-unit increase	1.059 (0.993–1.130)	0.082
	Respondent gender: woman vs. man	1.755 (1.158–2.658)	0.008
	Age, per year	1.005 (0.989–1.022)	0.508
	Farm size, per hectare	0.983 (0.961–1.007)	0.160
	Household size, per member	1.027 (0.922–1.143)	0.629
	Educational attainment, per one-category increase	0.906 (0.770–1.066)	0.236

Results are adjusted prevalence ratios with HC3 robust 95% confidence intervals from four separate modified Poisson models. FDCI and MFDI effects correspond to a 0.10-unit increase; HPDS corresponds to one additional food group. Educational attainment was modeled as an ordinal variable.

^a^Adjusted for FDCI, HPDS, respondent gender, farm size, and household size.

^b^Adjusted for FDCI, MFDI, respondent gender, farm size, and household size.

^c^Adjusted for FDCI, HPDS, respondent gender, farm size, and household size.

^d^Adjusted for FDCI, MFDI, respondent gender, age, farm size, household size, and educational attainment.

### Sensitivity analyses and model diagnostics

The association between production diversity and high market dependence was consistent across all major sensitivity analyses. Canton-clustered CR2 estimation produced an adjusted PR of 0.708 per additional food group (95% CI: 0.595–0.843; *p* = 0.002). Firth penalized logistic regression also showed an inverse association (OR: 0.584; 95% CI: 0.427–0.765; *p* < 0.001). In 2,000 bootstrap replications, the median PR was 0.701, with a percentile interval of 0.587–0.814, and the estimated association was inverse in 100% of valid replications (Supplementary Table 9).

The FDCI association was less uniform across estimators. The bootstrap median PR was 0.929 (percentile interval: 0.868–0.991), with an inverse direction in 98.9% of valid replications. The CR2 estimate retained the same point estimate but had a wider interval (PR: 0.933; 95% CI: 0.854–1.019; *p* = 0.107). Firth regression produced an OR of 0.881 (95% CI: 0.764–1.007; *p* = 0.063). The direction of the FDCI association was therefore consistent, although its precision decreased in the canton-clustered, penalized, and extended specifications (Supplementary Table 9).

Alternative FDCI definitions produced different magnitudes. The legacy truncated ratio had a PR of 0.878 per 0.10-unit increase (95% CI: 0.827–0.931; *p* < 0.001), whereas the Jaccard and Sørensen measures had PRs of 0.956 (95% CI: 0.895–1.021; *p* = 0.178) and 0.958 (95% CI: 0.894–1.026; *p* = 0.221), respectively. Combining pork with red meat produced an estimate similar to the primary exact-overlap definition (PR: 0.931; 95% CI: 0.872–0.993; *p* = 0.031; Supplementary Table 5 and Supplementary Figure 4).

In the first-stage residualization model, each additional food group produced was associated with a 0.0669-unit lower expected FDCI (95% CI: −0.0958 to −0.0380; *p* < 0.001), and HPDS explained 14.7% of the observed variation in the FDCI. The residualized FDCI was effectively uncorrelated with HPDS. In the modified Poisson sensitivity model, each 0.10-unit increase in the FDCI above the value expected from HPDS was associated with an adjusted prevalence ratio of 0.932 for high market dependence (95% CI: 0.865–1.004; *p* = 0.062). The magnitude and direction were similar to those of the primary simultaneous-adjustment model, although the residualized estimate was less precise. Retaining HPDS together with the residualized FDCI reproduced the primary FDCI estimate (aPR: 0.933; 95% CI: 0.874–0.996; *p* = 0.037), confirming the expected reparameterization equivalence (Supplementary Table 5).

In analyses restricted by the number of produced food groups, the FDCI PR was 0.890 among households producing at least two groups (95% CI: 0.793–1.000; *p* = 0.050) and 0.762 among households producing at least three groups (95% CI: 0.643–0.905; *p* = 0.002). These restrictions involved 94 and 65 households, respectively, and were treated as exploratory denominator-sensitivity analyses rather than formal tests of effect modification (Supplementary Table 5).

The high-market-dependence results were sensitive to the selected binary threshold. With the primary MFDI threshold of 0.75, both FDCI and production diversity were inversely associated with the outcome. With a threshold of 0.50, the estimates were attenuated and not statistically distinguishable from the null: PR 0.981 for FDCI (95% CI: 0.953–1.008; *p* = 0.169) and PR 0.983 for production diversity (95% CI: 0.940–1.028; *p* = 0.460). The inverse associations observed with the continuous MFDI provided complementary evidence without relying on a single internally defined cut-off (Supplementary Table 6).

Two observations exceeded the conventional Cook’s distance threshold of 4/n in the high-market-dependence model. Excluding household 90, household 2, or both households simultaneously did not change the direction or substantive interpretation of the estimates. Across these scenarios, the FDCI PR ranged from 0.921 to 0.934, compared with 0.933 in the complete sample, and the production-diversity PR ranged from 0.694 to 0.709, compared with 0.708 in the complete sample. Both households were retained in the primary analysis because no data error or eligibility violation was identified (Supplementary Figures 6, 7 and Supplementary Table 11).

Variance inflation factors did not indicate problematic multicollinearity. Models using natural splines did not improve the Akaike Information Criterion relative to the corresponding linear-term models, and the main estimates remained similar after excluding, winsorizing, or log-transforming the extreme farm-size value. Pearson dispersion ratios did not indicate overdispersion. Multicollinearity, linearity, and model diagnostics are presented in Supplementary Table 10, while influence and farm-size sensitivity analyses are provided in Supplementary Table 11.

## Discussion

This study showed that greater household production diversity was consistently associated with a broader range of food groups self-consumed from own production and with a lower prevalence of high market dependence. Exact farm-to-diet overlap was also inversely associated with market dependence in the primary adjusted model and the continuous-outcome analysis, although the magnitude of this association was modest and its precision decreased in several sensitivity specifications. By contrast, the exact-overlap Farm-to-Diet Coupling Index was not independently associated with food insecurity, market-dependent food vulnerability, or food-system buffer resilience. Food-acquisition pathways also differed substantially across groups. Vegetables, fish, and red meat were predominantly purchased, whereas cereals, grains, and legumes and fruits were more frequently produced than purchased. These findings indicate that production diversity, production–self-consumption overlap, and market acquisition represent related but distinct dimensions of the household food system.

The positive relationship between production diversity and self-consumed food-group diversity is consistent with findings from several smallholder settings. However, the evidence is not uniform. In a four-country analysis, Sibhatu et al. found positive associations in some settings but nonsignificant or inverse relationships where production diversity was already high; market access was generally more strongly associated with dietary diversity than additional farm diversification (23, 24). Similarly, a nationally representative study of farming households in Zambia found no evidence that agricultural production diversity was associated with household dietary diversity (25).

These inconsistencies may reflect differences in commercialization, market integration, seasonality, household resources, and the measurement of production and dietary diversity. Multicountry evidence suggests that the average association is modest and sensitive to the metric used (13), while research in the Ecuadorian Andes has also reported only limited relationships with dietary diversity and food insecurity (21). In our study, production diversity was associated with the number of food groups self-consumed from own production in both descriptive and adjusted analyses. Nevertheless, this finding should not be interpreted as evidence of improved overall dietary quality because the self-consumption proxy did not capture quantities, frequency, total intake, intrahousehold distribution, or nutrient adequacy.

The food-group results further illustrate why production, self-consumption, and purchase should be examined separately. Cereals, grains, and legumes and fruits showed high production prevalence and comparatively strong confirmed production–self-consumption overlap. Vegetables presented the largest positive purchase–production difference, followed by fish and red meat. Pork and poultry were more frequently produced than purchased, but only a limited proportion of producers reported consuming these groups from their own production. Previous research in Ecuador has similarly shown that foods obtained from own production, conventional markets, and social-economy sources contribute differently to household nutrient intake (22). Evidence from rural Ethiopia also indicates that purchased foods can make a substantial contribution to food consumption and dietary diversity throughout the agricultural year (26). Purchase predominance should not, however, be equated with nutritional inadequacy, because food quantity, quality, affordability, and nutrient intake were not measured.

The exact-overlap FDCI was developed to distinguish the breadth of household production from the proportion of the production portfolio that was also reported as self-consumed. Its inverse association with market dependence is conceptually consistent with non-separable agricultural household models, in which production and consumption decisions are jointly influenced by food preferences, transaction costs, market integration, household requirements, and risk (27). Decisions about what to produce, retain, sell, or purchase may therefore occur simultaneously rather than as independent stages. The FDCI operationalizes one observable aspect of this relationship by identifying exact correspondence between food groups reported in the household production and self-consumption inventories. It does not, however, establish the amount consumed, the economic value of retained production, or the temporal sequence connecting production with household food use.

The FDCI findings require a restrained interpretation. Its inverse association with market dependence was supported by the primary adjusted model, the continuous MFDI analysis, bootstrap resampling, and models excluding influential observations. However, the estimate became less precise when canton-clustered CR2 standard errors, Firth penalized logistic regression, broader adjustment sets, an alternative market-dependence threshold, and set-similarity definitions were used. Residualizing the FDCI with respect to production diversity preserved the magnitude and inverse direction of the association with market dependence, although the confidence interval included the null. This finding reinforces the interpretation of the FDCI association as modest and less precisely estimated than the association observed for production diversity. Methodological research has documented substantial heterogeneity in the construction and interpretation of production-diversity and market-related indicators, which limits direct comparison across studies (28). The exact-overlap FDCI should therefore be considered a complementary and locally constructed measure rather than a validated indicator or a superior predictor of household food-system outcomes. Its reliability, temporal stability, construct validity, and transportability require assessment in independent populations and across different agricultural seasons.

Production diversity showed a stronger and more consistent inverse association with high market dependence than exact farm-to-diet overlap. Because HPDS and FDCI were expressed in different units, this difference should not be interpreted as a direct comparison of effect magnitude. The findings nevertheless suggest that households producing more food groups may need to purchase a smaller proportion of the groups represented in their food-acquisition portfolio. This does not imply that market participation is harmful or that households should attempt to produce every food they consume. Systematic-review and multicountry evidence indicates that market access can support dietary diversity and may be more important than additional diversification at the individual-farm level (13, 14). Markets can expand food options and provide products that households cannot efficiently produce or obtain throughout the year. Production and markets should therefore be understood as complementary rather than competing pathways.

Exact farm-to-diet overlap was not independently associated with high food insecurity or with market-dependent food vulnerability. These null findings delimit the explanatory scope of the FDCI. A household may retain a large proportion of its production for self-consumption and still experience food insecurity if production quantities are insufficient, the food portfolio does not meet household needs, income is unstable, or access to complementary foods is constrained. Research in rural Ecuador has identified household size, education, food expenditure, cultivated area, and other socioeconomic characteristics as correlates of dietary diversity (20). Multicountry evidence also indicates that smallholder food security varies with rainfall conditions and access to household resources (29). Production–self-consumption overlap should therefore not be used as a stand-alone measure of food security or vulnerability, which remain multidimensional conditions shaped by economic, environmental, and household-level factors.

Neither the FDCI nor the MFDI was independently associated with low food-system buffer resilience in the core model. The clearest association was a higher prevalence of low buffer resilience among households represented by woman respondents. This result requires particular caution because respondent gender did not necessarily represent household headship, ownership of productive resources, control of income, or authority over food-related decisions. Women also constituted a relatively small proportion of respondents. Previous research indicates that specific dimensions of women’s empowerment, including autonomy and participation in productive decisions, may be associated with food-group consumption, but the magnitude and direction of these relationships vary across settings (30). Respondent gender alone cannot capture these processes. The association should consequently be regarded as hypothesis-generating and investigated using direct measures of land access, asset ownership, decision-making, income control, and labor responsibilities.

Commercial crops were common in the broader production audit but were excluded from the primary dietary food-group indicators because their relationship with direct household food use could not be interpreted in the same way as that of food crops and animal-source foods. Their exclusion prevented commercially oriented production from artificially increasing the HPDS and FDCI. Commercial production may nevertheless influence household diets indirectly through income and food purchases. Evidence from smallholder households in Kenya suggests that agricultural commercialization can improve food security and dietary quality through income-related pathways, although its effects depend on market access, gendered income control, productivity, and possible changes in own-produced food consumption (31). Because the present study did not measure crop-specific sales, profitability, agricultural income, or allocation of earnings, it was not possible to determine whether commercial orientation improved food access or increased exposure to market fluctuations.

These findings have implications for nutrition-sensitive agricultural programs. Monitoring only the number of crops or food groups produced may overlook whether those products are retained, sold, exchanged, or used within the household. Production, self-consumption, commercialization, and market purchase should be treated as interconnected but analytically distinct pathways. In Los Ríos, programs may benefit from examining why vegetables and several animal-source food groups were predominantly purchased while simultaneously supporting reliable markets, income stability, and household financial capacity. Evidence suggests that market functioning and food availability at local and regional levels may be at least as important as increasing diversity on every individual farm (13, 14). Experimental evidence also indicates that nutrition-sensitive strategies may be more effective when agricultural learning is combined with nutrition education, agroecological practices, and attention to household decision-making (32). The present results do not support replacing markets with own production or treating diversification as an isolated intervention.

This study has several strengths. Production, self-consumption from own production, and purchase were reconstructed using a common food-group classification, allowing household-level overlap to be measured rather than inferred from separate diversity counts. Food-group inconsistencies were audited, and self-consumption responses without matching production records were not automatically classified as confirmed overlap. The statistical analysis estimated prevalence ratios for common outcomes and applied parsimonious, outcome-specific adjustment sets. Continuous-outcome models, alternative FDCI definitions, bootstrap resampling, penalized logistic regression, canton-clustered inference, denominator restrictions, alternative food classifications, and influence analyses were used to evaluate the stability of the findings. These procedures do not establish external validity, but they make the dependence of the results on analytical assumptions and operational definitions more transparent.

Several limitations should be considered. The cross-sectional design precludes establishing temporal order or causal relationships among production, self-consumption, market acquisition, food insecurity, and resilience. Household recruitment was non-probabilistic, and the results cannot be considered representative of all smallholder households in Los Ríos or its individual cantons. The sample size was modest, and cluster-robust inference relied on only ten cantons, limiting the precision of those estimates. Although core models were kept parsimonious, some estimates remained sensitive to alternative adjustment and variance specifications. Residual confounding is also possible because the survey did not comprehensively measure income, food prices, distance to markets, crop-specific profitability, land tenure, household decision-making, social protection, or recent exposure to climatic and economic shocks.

Measurement limitations are also important. All variables were self-reported, and the questionnaire did not specify a standardized dietary recall period. The Self-consumed Food Group Diversity Proxy should therefore not be interpreted as a validated dietary diversity score or as a measure of usual dietary intake. Reconstruction of free-text responses may have introduced classification error despite predefined harmonization and auditing procedures. Because the FDCI uses the number of produced food groups as its denominator, it may be particularly sensitive among households with low production diversity, where a single concordant or discordant group can substantially change the index. The restricted-denominator analyses should therefore be interpreted as exploratory assessments of this measurement property rather than as evidence of effect modification. The FDCI, HMDFVI, and Food System Buffer Resilience Index were constructed for this study and require independent validation. Median-based thresholds represented relative comparisons within the sample rather than established population cut-offs. Finally, data collected during a single month could not capture harvest cycles, food-price changes, or seasonal variation in food acquisition. Longitudinal evidence demonstrates that dietary diversity and the contribution of production and markets can change across agricultural seasons (26, 33).

## Conclusion

Greater household production diversity was associated with a broader range of food groups self-consumed from own production and with lower market dependence among smallholder farming households in Los Ríos, Ecuador. Production, self-consumption, and purchase nevertheless followed heterogeneous pathways across food groups. Vegetables, fish, and red meat were obtained predominantly through purchase, whereas cereals, grains, and legumes and fruits were more frequently produced than purchased. These patterns show that agricultural participation does not eliminate the need for market acquisition and that production diversity should not be treated as equivalent to household dietary diversity or food security.

The exact-overlap Farm-to-Diet Coupling Index was inversely associated with market dependence in the primary adjusted and continuous-outcome analyses. However, the magnitude of the association was modest and its precision decreased under several alternative specifications. No consistent independent associations were identified between the FDCI and food insecurity, market-dependent food vulnerability, or food-system buffer resilience. The FDCI should therefore be interpreted as a complementary operational measure of production–self-consumption correspondence rather than as a validated or superior indicator of household food-system outcomes.

Agricultural and nutrition-sensitive programs should examine not only the diversity of household production, but also which food groups are retained for household use, which are commercialized, and which continue to be obtained through markets. The findings do not support replacing market acquisition with own production or assuming that production diversification alone will reduce food insecurity. Future studies should validate the FDCI in independent populations using probability-based and longitudinal designs, repeated seasonal measurements, standardized dietary assessment, and detailed information on production quantities, food sales, agricultural income, expenditures, prices, market access, and household decision-making. Definitions and operationalization details for the study indicators are provided in Supplementary Table 14.

## Data Availability

The de-identified data supporting the findings of this study are available from the corresponding author upon reasonable request and subject to applicable ethical and institutional requirements. The definitions of the study indicators, diagnostic results, and sensitivity analyses are provided in the Supplementary material. Requests to access the datasets should be directed to RZ-V, razambrano@ecotec.edu.ec.
