# Supplementary material for: Associations of the exact-overlap Farm-to-Diet Coupling Index and production diversity with market dependence among smallholder farming households in Los Ríos, Ecuador: a cross-sectional study
Source: Front Nutr. 2026 Sep 8;13:1923220. doi: 10.3389/fnut.2026.1923220 (PMC13593439; doi:10.3389/fnut.2026.1923220)
Supplement: Supplementary file 1 [file Table_1.docx]

# Supplementary materials

# Index of supplementary materials

**Supplementary Table S1.** Spearman correlation matrix of primary household food-system indicators

**Supplementary Table S2.** Original survey questions and analytical interpretation

**Supplementary Table S3.** Preliminary audit of the Farm-to-Diet Coupling Index

**Supplementary Table S4.** Self-consumption records without a matching production record

**Supplementary Table S5.** Sensitivity of the market-dependence association to FDCI definition and denominator restrictions

**Supplementary Table S6.** Alternative market-dependence threshold and continuous-outcome analyses

**Supplementary Table S7.** Production diversity and the Self-consumed Food Group Diversity Proxy: count-model results

**Supplementary Table S8.** Analytical flow, model stability, and events per estimated parameter

**Supplementary Table S9.** Firth, canton-clustered CR2, and bootstrap sensitivity analyses

**Supplementary Table S10.** Multicollinearity, model diagnostics, and linearity assessment

**Supplementary Table S11.** Influence and farm-size sensitivity analyses for the market-dependence model

**Supplementary Table S12.** Missingness and reliability evaluation

**Supplementary Table S13.** Evaluation of the formative vulnerability and buffer-resilience indices

**Supplementary Table S14.** Indicator dictionary

**Supplementary Figure S1.** Spearman correlation structure of primary food-system indicators

**Supplementary Figure S2.** Distribution of alternative farm-to-diet coupling definitions

**Supplementary Figure S3.** Legacy count ratio versus exact production–self-consumption overlap

**Supplementary Figure S4.** Sensitivity of the market-dependence association to FDCI definition

**Supplementary Figure S5.** Missingness in variables used in the main models

**Supplementary Figure S6.** Cook's distance across core modified Poisson models

**Supplementary Figure S7.** Sensitivity of the market-dependence model to influential observations

**Supplementary Table S1.** Spearman correlation matrix of primary household food-system indicators

| **Indicator** | **HPDS** | **SCFGD proxy** | **Exact-overlap FDCI** | **MFDI** | **Food insecurity** | **Agroecological transition** | **Financial resilience** |
| --- | --- | --- | --- | --- | --- | --- | --- |
| HPDS | 1.00 | 0.64 | -0.41 | -0.27 | 0.18 | 0.07 | -0.07 |
| SCFGD proxy | 0.64 | 1.00 | 0.19 | -0.51 | 0.10 | 0.06 | 0.03 |
| Exact-overlap FDCI | -0.41 | 0.19 | 1.00 | -0.08 | -0.09 | 0.05 | 0.15 |
| MFDI | -0.27 | -0.51 | -0.08 | 1.00 | 0.15 | -0.06 | 0.13 |
| Food insecurity | 0.18 | 0.10 | -0.09 | 0.15 | 1.00 | -0.15 | -0.15 |
| Agroecological transition | 0.07 | 0.06 | 0.05 | -0.06 | -0.15 | 1.00 | 0.37 |
| Financial resilience | -0.07 | 0.03 | 0.15 | 0.13 | -0.15 | 0.37 | 1.00 |

**Note.** Values are Spearman rank-correlation coefficients. SCFGD proxy, Self-consumed Food Group Diversity Proxy; FDCI, Farm-to-Diet Coupling Index; HPDS, Household Production Diversity Score; MFDI, Market Food Dependency Index. Correlations are descriptive and do not imply causal relationships.

**Supplementary Table S2.** Original survey questions and analytical interpretation

| **Domain** | **Original questionnaire item** | **Recall framework** | **Analytical interpretation** |
| --- | --- | --- | --- |
| Agricultural self-consumption | ¿Qué productos agrícolas provenientes de su producción consumen en su hogar? | No explicit recall window in the available questionnaire text | Foods reported as consumed from the household's own agricultural production |
| Livestock self-consumption | ¿Qué productos pecuarios provenientes de su producción consumen en su hogar? | No explicit recall window in the available questionnaire text | Foods reported as consumed from the household's own livestock production |
| Agricultural purchase | ¿Qué productos agrícolas tiene que comprar para su sustento diario? | Daily sustenance wording; no formal 24-hour or 7-day recall | Agricultural foods reported as requiring purchase |
| Livestock purchase | ¿Qué productos pecuarios tiene que comprar para su sustento diario? | Daily sustenance wording; no formal 24-hour or 7-day recall | Livestock foods reported as requiring purchase |

**Note.** The questions were open ended and permitted multiple products within a response. The questionnaire did not apply a standardized dietary recall instrument; self-consumption was therefore treated as a household food-group proxy rather than as a validated dietary diversity measure.

**Supplementary Table S3.** Preliminary audit of the Farm-to-Diet Coupling Index

**Panel A. Overall audit summary**

| **Audit measure** | **Value** |
| --- | --- |
| Households in analytical dataset | 133 |
| Households with HPDS = 0 | 2 |
| Households with calculable raw count ratio | 131 |
| Raw count ratio >1, n | 6 |
| Raw count ratio >1, % | 4.6 |
| Raw count ratio, median (IQR) | 0.60 (0.50–1.00) |
| Maximum raw count ratio | 3 |
| Exact-overlap FDCI, median (IQR) | 0.50 (0.40–1.00) |
| Households with ≥1 self-consumed group without matching production, n | 18 |
| Households with ≥1 discordance, % | 13.5 |

**Panel B. Audit according to household production diversity**

| **HPDS category** | **n** | **Raw ratio available** | **Raw ratio >1, n** | **Raw ratio >1, %** | **Exact FDCI median** | **Q1** | **Q3** |
| --- | --- | --- | --- | --- | --- | --- | --- |
| 0 groups | 2 | 0 | 0 | — | — | — | — |
| 1 group | 32 | 32 | 3 | 9.4 | 1 | 1 | 1 |
| 2 groups | 32 | 32 | 2 | 6.2 | 0.50 | 0.50 | 0.50 |
| 3 groups | 22 | 22 | 1 | 4.5 | 0.67 | 0.33 | 0.92 |
| 4 or more groups | 45 | 45 | 0 | 0.0 | 0.50 | 0.33 | 0.60 |

**Note.** The raw count ratio divided the number of self-consumed groups by the number of produced groups without verifying food-group correspondence. The primary exact-overlap FDCI counted only groups reported as both produced and self-consumed by the same household. Households with HPDS = 0 had an undefined ratio-based FDCI.

**Supplementary Table S4.** Self-consumption records without a matching production record

**Panel A. Discordances by food group**

| **Food group** | **Household-group discordances** | **Affected households** | **Also purchased** |
| --- | --- | --- | --- |
| Tubers and roots | 6 | 6 | 2 |
| Fish | 4 | 4 | 1 |
| Fruits | 4 | 4 | 0 |
| Pork | 4 | 4 | 1 |
| White meat/poultry | 3 | 3 | 0 |
| Cereals, grains, and legumes | 2 | 2 | 1 |
| Vegetables | 2 | 2 | 1 |
| Dairy products | 1 | 1 | 0 |
| Eggs | 1 | 1 | 0 |

**Panel B. Household-level discordance summary**

| **Record ID** | **Canton** | **Respondent gender** | **HPDS** | **SCFGD proxy** | **Raw ratio** | **Exact FDCI** | **Self-consumed groups without matching production** |
| --- | --- | --- | --- | --- | --- | --- | --- |
| 2 | Montalvo | Women | 1 | 3 | 3 | 0 | Fruits; Pork; White meat/poultry |
| 104 | Vinces | Women | 2 | 2 | 1 | 0 | Cereals, grains, and legumes; White meat/poultry |
| 30 | Ventanas | Women | 2 | 3 | 1.50 | 0.50 | Fruits; Tubers and roots |
| 37 | Babahoyo | Men | 2 | 2 | 1 | 0 | Eggs; Pork |
| 43 | Palenque | Men | 5 | 4 | 0.80 | 0.40 | Fish; Tubers and roots |
| 45 | Babahoyo | Men | 2 | 3 | 1.50 | 0.50 | Fruits; Tubers and roots |
| 66 | Mocache | Men | 3 | 5 | 1.67 | 1 | Pork; White meat/poultry |
| 83 | Ventanas | Women | 1 | 3 | 3 | 1 | Tubers and roots; Vegetables |
| 125 | Valencia | Women | 3 | 2 | 0.67 | 0.33 | Vegetables |
| 133 | Pueblo Viejo | Men | 4 | 2 | 0.50 | 0.25 | Cereals, grains, and legumes |
| 33 | Baba | Men | 4 | 2 | 0.50 | 0.25 | Fish |
| 47 | Ventanas | Men | 2 | 1 | 0.50 | 0 | Fruits |
| 63 | Baba | Men | 3 | 3 | 1 | 0.67 | Pork |
| 7 | Urdaneta | Women | 2 | 2 | 1 | 0.50 | Dairy products |
| 84 | Mocache | Men | 5 | 3 | 0.60 | 0.40 | Tubers and roots |
| 92 | Ventanas | Men | 6 | 3 | 0.50 | 0.33 | Fish |
| 94 | Vinces | Men | 1 | 2 | 2 | 1 | Fish |
| 96 | Babahoyo | Men | 5 | 3 | 0.60 | 0.40 | Tubers and roots |

**Note.** Discordant self-consumption records were retained as reported but were not counted in the numerator of the exact-overlap FDCI. Record identifiers are analytical IDs and do not contain personal identifiers.

**Supplementary Table S5.** Sensitivity of the market-dependence association to FDCI definition and denominator restrictions

**Panel A. Alternative FDCI definitions and food-group classification**

| **FDCI definition** | **n** | **aPR per 0.10 increase** | **95% CI** | **p value** |
| --- | --- | --- | --- | --- |
| Exact overlap | 124 | 0.933 | 0.874–0.996 | 0.037 |
| Legacy truncated ratio | 124 | 0.878 | 0.827–0.931 | <0.001 |
| Jaccard similarity | 124 | 0.956 | 0.895–1.021 | 0.178 |
| Sørensen similarity | 124 | 0.958 | 0.894–1.026 | 0.221 |
| Exact overlap, pork combined with red meat | 124 | 0.931 | 0.872–0.993 | 0.031 |

**Panel B. Denominator restrictions according to minimum production diversity**

| **Analytical restriction** | **n** | **aPR per 0.10 increase** | **95% CI** | **p value** |
| --- | --- | --- | --- | --- |
| HPDS ≥1 | 124 | 0.933 | 0.874–0.996 | 0.037 |
| HPDS ≥2 | 94 | 0.890 | 0.793–1.000 | 0.050 |
| HPDS ≥3 | 65 | 0.762 | 0.643–0.905 | 0.002 |

**Panel C. Residualized FDCI sensitivity analysis**

| **Analysis** | **n** | **Events** | **aPR per 0.10 increase** | **95% CI** | **p value** |
| --- | --- | --- | --- | --- | --- |
| Primary exact-overlap FDCI, adjusted simultaneously for HPDS | 124 | 49 | 0.933 | 0.874–0.996 | 0.037 |
| Residualized exact-overlap FDCI above the value expected from HPDS | 124 | 49 | 0.932 | 0.865–1.004 | 0.062 |
| Residualized exact-overlap FDCI with HPDS retained | 124 | 49 | 0.933 | 0.874–0.996 | 0.037 |

**Note.** Adjusted prevalence ratios were estimated for high market dependence using the corresponding FDCI definition. Restricted-denominator analyses were exploratory and were not interpreted as evidence of effect modification. The exact-overlap FDCI was residualized using an ordinary least-squares regression on HPDS. Residuals were rescaled so that estimates corresponded to a 0.10-unit increase in coupling above or below the value expected from production diversity. The residualized-only model was adjusted for respondent gender, farm size, and household size. The third specification retained HPDS as a reparameterization check and produced predictions equivalent to those of the primary simultaneous-adjustment model.

**Supplementary Table S6.** Alternative market-dependence threshold and continuous-outcome analyses

**Panel A. Alternative threshold for high market dependence**

| **Outcome definition** | **Exposure** | **n** | **aPR** | **95% CI** | **p value** |
| --- | --- | --- | --- | --- | --- |
| MFDI ≥0.75 | Exact-overlap FDCI, per 0.10 increase | 124 | 0.933 | 0.874–0.996 | 0.037 |
| MFDI ≥0.75 | HPDS, per food group | 124 | 0.708 | 0.603–0.832 | <0.001 |
| MFDI ≥0.50 | Exact-overlap FDCI, per 0.10 increase | 124 | 0.981 | 0.953–1.008 | 0.169 |
| MFDI ≥0.50 | HPDS, per food group | 124 | 0.983 | 0.940–1.028 | 0.460 |

**Panel B. Fractional-logit continuous-outcome models**

| **Continuous outcome** | **Predictor** | **n** | **Coefficient** | **95% CI** | **p value** |
| --- | --- | --- | --- | --- | --- |
| Market Food Dependency Index | Intercept | 124 | 1.651 | 0.515–2.788 | 0.004 |
| Market Food Dependency Index | Exact-overlap FDCI, per 0.10 | 124 | -0.096 | -0.185–-0.007 | 0.035 |
| Market Food Dependency Index | HPDS, per group | 124 | -0.196 | -0.317–-0.074 | 0.002 |
| Market Food Dependency Index | Woman respondent | 124 | -0.467 | -1.138–0.204 | 0.173 |
| Market Food Dependency Index | Farm size, ha | 124 | 0.003 | -0.007–0.013 | 0.583 |
| Market Food Dependency Index | Household size | 124 | 0.038 | -0.109–0.186 | 0.611 |
| Food insecurity index | Intercept | 124 | -1.503 | -2.754–-0.251 | 0.019 |
| Food insecurity index | Exact-overlap FDCI, per 0.10 | 124 | -0.017 | -0.097–0.063 | 0.673 |
| Food insecurity index | MFDI, per 0.10 | 124 | 0.081 | -0.008–0.171 | 0.074 |
| Food insecurity index | Woman respondent | 124 | 0.464 | -0.147–1.075 | 0.137 |
| Food insecurity index | Farm size, ha | 124 | -0.008 | -0.017–0.001 | 0.091 |
| Food insecurity index | Household size | 124 | 0.083 | -0.076–0.241 | 0.305 |
| Market-dependent food vulnerability index | Intercept | 124 | 0.287 | -0.311–0.885 | 0.347 |
| Market-dependent food vulnerability index | Exact-overlap FDCI, per 0.10 | 124 | -0.039 | -0.082–0.004 | 0.077 |
| Market-dependent food vulnerability index | HPDS, per group | 124 | -0.030 | -0.099–0.038 | 0.384 |
| Market-dependent food vulnerability index | Woman respondent | 124 | 0.039 | -0.327–0.406 | 0.834 |
| Market-dependent food vulnerability index | Farm size, ha | 124 | -0.002 | -0.007–0.002 | 0.344 |
| Market-dependent food vulnerability index | Household size | 124 | 0.013 | -0.067–0.093 | 0.751 |
| Food system buffer-resilience score | Intercept | 122 | 0.325 | -0.435–1.085 | 0.402 |
| Food system buffer-resilience score | Exact-overlap FDCI, per 0.10 | 122 | 0.020 | -0.026–0.065 | 0.393 |
| Food system buffer-resilience score | MFDI, per 0.10 | 122 | -0.020 | -0.065–0.025 | 0.385 |
| Food system buffer-resilience score | Woman respondent | 122 | -0.380 | -0.739–-0.021 | 0.038 |
| Food system buffer-resilience score | Age, years | 122 | -0.005 | -0.014–0.005 | 0.351 |
| Food system buffer-resilience score | Farm size, ha | 122 | 0.006 | -4.34e-04–0.012 | 0.069 |
| Food system buffer-resilience score | Household size | 122 | -0.004 | -0.087–0.079 | 0.932 |
| Food system buffer-resilience score | Educational attainment | 122 | 0.012 | -0.087–0.111 | 0.815 |

**Panel C. Average marginal effect of exact-overlap FDCI**

| **Continuous outcome** | **Average marginal effect per 0.10 increase** | **95% CI** | **p value** |
| --- | --- | --- | --- |
| Market Food Dependency Index | -0.0215 | -0.0409–-0.0020 | 0.030 |
| Food insecurity index | -0.0038 | -0.0212–0.0137 | 0.673 |
| Market-dependent food vulnerability index | -0.0097 | -0.0205–0.0010 | 0.075 |
| Food system buffer-resilience score | 0.0049 | -0.0063–0.0160 | 0.391 |

**Note.** Coefficients are on the fractional-logit linear-predictor scale. Average marginal effects are expressed on the response scale as the expected absolute change in the outcome associated with a 0.10-unit increase in the FDCI.

**Supplementary Table S7.** Production diversity and the Self-consumed Food Group Diversity Proxy: count-model results

**Panel A. Adjusted count model**

| **Predictor** | **Rate ratio** | **95% CI** | **p value** |
| --- | --- | --- | --- |
| Intercept | 1.187 | 0.893–1.577 | 0.238 |
| HPDS, per food group | 1.236 | 1.191–1.283 | <0.001 |
| Woman respondent | 1.119 | 0.896–1.398 | 0.321 |
| Farm size, ha | 0.997 | 0.989–1.005 | 0.407 |
| Household size | 0.944 | 0.891–0.999 | 0.047 |

**Panel B. Model comparison**

| **Count-model specification** | **AIC** | **Pearson dispersion ratio** |
| --- | --- | --- |
| Poisson | 363.64 | 0.420 |
| Negative binomial | 365.64 | — |

**Note.** The outcome was the Self-consumed Food Group Diversity Proxy. The Poisson specification was retained because it had the lower AIC and did not show evidence of overdispersion. HC3 robust standard errors were used for inference.

**Supplementary Table S8.** Analytical flow, model stability, and events per estimated parameter

**Panel A. Model stability and events per estimated parameter**

| **Outcome** | **Specification** | **n** | **Events** | **Non-events** | **Prevalence, %** | **Parameters** | **Events/parameter** | **AIC** |
| --- | --- | --- | --- | --- | --- | --- | --- | --- |
| High market dependence | Crude | 127 | 51 | 76 | 40.2 | 1 | 51 | 199.06 |
| High market dependence | Core | 124 | 49 | 75 | 39.5 | 5 | 9.80 | 188.71 |
| High market dependence | Extended | 118 | 46 | 72 | 39.0 | 8 | 5.75 | 183.16 |
| High food insecurity | Crude | 127 | 67 | 60 | 52.8 | 2 | 33.50 | 224.54 |
| High food insecurity | Core | 124 | 66 | 58 | 53.2 | 5 | 13.20 | 223.68 |
| High food insecurity | Extended | 122 | 64 | 58 | 52.5 | 7 | 9.14 | 219.93 |
| High market-dependent vulnerability | Crude | 127 | 63 | 64 | 49.6 | 2 | 31.50 | 218.89 |
| High market-dependent vulnerability | Core | 124 | 61 | 63 | 49.2 | 5 | 12.20 | 216.99 |
| High market-dependent vulnerability | Extended | 119 | 58 | 61 | 48.7 | 7 | 8.29 | 206.40 |
| Low food system buffer resilience | Crude | 125 | 64 | 61 | 51.2 | 2 | 32 | 218.49 |
| Low food system buffer resilience | Core | 122 | 62 | 60 | 50.8 | 7 | 8.86 | 215.93 |

**Panel B. Analytical flow**

| **Analytical step** | **n** |
| --- | --- |
| Households imported from analytical database | 133 |
| Unique households after harmonisation | 133 |
| Households producing at least one food group | 131 |
| Households with exact-overlap FDCI available | 131 |
| Complete cases: High market dependence | 124 |
| Complete cases: High food insecurity | 124 |
| Complete cases: High market-dependent vulnerability | 124 |
| Complete cases: Low food system buffer resilience | 122 |

**Note.** Extended models had fewer events per parameter and were therefore treated as sensitivity analyses. Complete-case sample sizes varied according to the variables included in each model.

**Supplementary Table S9.** Firth, canton-clustered CR2, and bootstrap sensitivity analyses

**Panel A. Firth penalized logistic regression**

| **Outcome** | **Predictor** | **Odds ratio** | **95% CI** | **p value** |
| --- | --- | --- | --- | --- |
| High market dependence | Intercept | 9.616 | 1.420–76.744 | 0.020 |
| High market dependence | Exact-overlap FDCI, per 0.10 | 0.881 | 0.764–1.007 | 0.063 |
| High market dependence | HPDS, per group | 0.584 | 0.427–0.765 | <0.001 |
| High market dependence | Woman respondent | 0.716 | 0.255–1.915 | 0.510 |
| High market dependence | Farm size, ha | 1.001 | 0.988–1.023 | 0.832 |
| High market dependence | Household size | 0.935 | 0.725–1.191 | 0.591 |
| High food insecurity | Intercept | 0.518 | 0.084–3.014 | 0.466 |
| High food insecurity | Exact-overlap FDCI, per 0.10 | 0.961 | 0.852–1.082 | 0.512 |
| High food insecurity | MFDI, per 0.10 | 1.083 | 0.955–1.235 | 0.214 |
| High food insecurity | Woman respondent | 1.129 | 0.453–2.884 | 0.795 |
| High food insecurity | Farm size, ha | 0.991 | 0.942–1.006 | 0.253 |
| High food insecurity | Household size | 1.133 | 0.909–1.429 | 0.268 |
| High market-dependent vulnerability | Intercept | 2.478 | 0.432–15.243 | 0.311 |
| High market-dependent vulnerability | Exact-overlap FDCI, per 0.10 | 0.900 | 0.788–1.022 | 0.105 |
| High market-dependent vulnerability | HPDS, per group | 0.923 | 0.739–1.145 | 0.469 |
| High market-dependent vulnerability | Woman respondent | 1.907 | 0.759–5.045 | 0.171 |
| High market-dependent vulnerability | Farm size, ha | 0.992 | 0.955–1.007 | 0.325 |
| High market-dependent vulnerability | Household size | 0.982 | 0.784–1.224 | 0.869 |
| Low food system buffer resilience | Intercept | 0.268 | 0.021–3.317 | 0.304 |
| Low food system buffer resilience | Exact-overlap FDCI, per 0.10 | 1.055 | 0.930–1.200 | 0.407 |
| Low food system buffer resilience | MFDI, per 0.10 | 1.130 | 0.990–1.300 | 0.070 |
| Low food system buffer resilience | Woman respondent | 3.943 | 1.417–12.375 | 0.008 |
| Low food system buffer resilience | Age, years | 1.009 | 0.979–1.041 | 0.570 |
| Low food system buffer resilience | Farm size, ha | 0.992 | 0.929–1.009 | 0.446 |
| Low food system buffer resilience | Household size | 1.030 | 0.817–1.301 | 0.801 |
| Low food system buffer resilience | Educational attainment | 0.798 | 0.576–1.105 | 0.164 |

**Panel B. CR2 small-sample-corrected cluster-robust inference by canton**

| **Outcome** | **Predictor** | **n** | **Clusters** | **aPR** | **95% CI** | **p value** |
| --- | --- | --- | --- | --- | --- | --- |
| High market dependence | Intercept | 124 | 10 | 1.961 | 0.331–11.621 | 0.414 |
| High market dependence | Exact-overlap FDCI, per 0.10 | 124 | 10 | 0.933 | 0.854–1.019 | 0.107 |
| High market dependence | HPDS, per group | 124 | 10 | 0.708 | 0.595–0.843 | 0.002 |
| High market dependence | Woman respondent | 124 | 10 | 0.805 | 0.370–1.751 | 0.543 |
| High market dependence | Farm size, ha | 124 | 10 | 1.001 | 0.991–1.011 | 0.778 |
| High market dependence | Household size | 124 | 10 | 0.950 | 0.762–1.184 | 0.612 |
| High food insecurity | Intercept | 124 | 10 | 0.371 | 0.123–1.122 | 0.073 |
| High food insecurity | Exact-overlap FDCI, per 0.10 | 124 | 10 | 0.982 | 0.896–1.076 | 0.665 |
| High food insecurity | MFDI, per 0.10 | 124 | 10 | 1.040 | 0.970–1.116 | 0.237 |
| High food insecurity | Woman respondent | 124 | 10 | 1.033 | 0.672–1.588 | 0.870 |
| High food insecurity | Farm size, ha | 124 | 10 | 0.987 | 0.973–1.002 | 0.074 |
| High food insecurity | Household size | 124 | 10 | 1.066 | 0.961–1.181 | 0.196 |
| High market-dependent vulnerability | Intercept | 124 | 10 | 0.761 | 0.283–2.044 | 0.548 |
| High market-dependent vulnerability | Exact-overlap FDCI, per 0.10 | 124 | 10 | 0.953 | 0.857–1.060 | 0.330 |
| High market-dependent vulnerability | HPDS, per group | 124 | 10 | 0.968 | 0.797–1.176 | 0.715 |
| High market-dependent vulnerability | Woman respondent | 124 | 10 | 1.314 | 0.813–2.122 | 0.230 |
| High market-dependent vulnerability | Farm size, ha | 124 | 10 | 0.990 | 0.974–1.006 | 0.193 |
| High market-dependent vulnerability | Household size | 124 | 10 | 0.994 | 0.897–1.101 | 0.897 |
| Low food system buffer resilience | Intercept | 122 | 10 | 0.247 | 0.036–1.668 | 0.132 |
| Low food system buffer resilience | Exact-overlap FDCI, per 0.10 | 122 | 10 | 1.025 | 0.978–1.075 | 0.263 |
| Low food system buffer resilience | MFDI, per 0.10 | 122 | 10 | 1.059 | 0.967–1.160 | 0.187 |
| Low food system buffer resilience | Woman respondent | 122 | 10 | 1.755 | 0.962–3.200 | 0.063 |
| Low food system buffer resilience | Age, years | 122 | 10 | 1.005 | 0.983–1.029 | 0.603 |
| Low food system buffer resilience | Farm size, ha | 122 | 10 | 0.983 | 0.962–1.005 | 0.121 |
| Low food system buffer resilience | Household size | 122 | 10 | 1.027 | 0.869–1.214 | 0.728 |
| Low food system buffer resilience | Educational attainment | 122 | 10 | 0.906 | 0.817–1.006 | 0.061 |

**Panel C. Nonparametric bootstrap sensitivity**

| **Outcome** | **Predictor** | **Valid replications** | **Median ratio** | **Bootstrap 95% interval** | **Direction stability, %** |
| --- | --- | --- | --- | --- | --- |
| High market dependence | Exact-overlap FDCI, per 0.10 | 2000 | 0.929 | 0.868–0.991 | 98.9 |
| High market dependence | HPDS, per group | 2000 | 0.701 | 0.587–0.814 | 100.0 |
| High food insecurity | Exact-overlap FDCI, per 0.10 | 2000 | 0.980 | 0.930–1.036 | 77.5 |
| High food insecurity | MFDI, per 0.10 | 2000 | 1.038 | 0.979–1.112 | 88.2 |
| High market-dependent vulnerability | Exact-overlap FDCI, per 0.10 | 2000 | 0.950 | 0.894–1.008 | 95.6 |
| High market-dependent vulnerability | HPDS, per group | 2000 | 0.966 | 0.863–1.086 | 72.7 |
| Low food system buffer resilience | Exact-overlap FDCI, per 0.10 | 2000 | 1.026 | 0.964–1.090 | 77.9 |
| Low food system buffer resilience | MFDI, per 0.10 | 2000 | 1.059 | 1.003–1.138 | 98.1 |

**Note.** Firth models report odds ratios and are provided only as penalized sensitivity analyses. CR2 models used ten canton clusters and should be interpreted cautiously. Bootstrap direction stability is the percentage of valid replications retaining the direction of the full-sample estimate.

**Supplementary Table S10.** Multicollinearity, model diagnostics, and linearity assessment

**Panel A. Variance inflation factors**

| **Model** | **Predictor** | **VIF** |
| --- | --- | --- |
| market_core | Exact-overlap FDCI, per 0.10 | 1.30 |
| market_core | HPDS, per group | 1.17 |
| market_core | gender | 1.07 |
| market_core | Farm size, ha | 1.03 |
| market_core | Household size | 1.14 |
| food_insecurity_core | Exact-overlap FDCI, per 0.10 | 1.12 |
| food_insecurity_core | MFDI, per 0.10 | 1.03 |
| food_insecurity_core | gender | 1.03 |
| food_insecurity_core | Farm size, ha | 1.02 |
| food_insecurity_core | Household size | 1.10 |
| vulnerability_core | Exact-overlap FDCI, per 0.10 | 1.21 |
| vulnerability_core | HPDS, per group | 1.16 |
| vulnerability_core | gender | 1.07 |
| vulnerability_core | Farm size, ha | 1.03 |
| vulnerability_core | Household size | 1.14 |
| buffer_core | Exact-overlap FDCI, per 0.10 | 1.18 |
| buffer_core | MFDI, per 0.10 | 1.03 |
| buffer_core | gender | 1.16 |
| buffer_core | Age, years | 1.51 |
| buffer_core | Farm size, ha | 1.11 |
| buffer_core | Household size | 1.15 |
| buffer_core | Educational attainment | 1.50 |

**Panel B. Overall model diagnostics**

| **Model** | **n** | **Pearson dispersion** | **Maximum Cook distance** | **4/n threshold** | **Cook >4/n** | **Maximum leverage** | **High leverage** |
| --- | --- | --- | --- | --- | --- | --- | --- |
| market_core | 124 | 0.592 | 0.481 | 0.032 | 2 | 0.872 | 6 |
| food_insecurity_core | 124 | 0.486 | 0.020 | 0.032 | 0 | 0.299 | 9 |
| vulnerability_core | 124 | 0.525 | 0.028 | 0.032 | 0 | 0.448 | 9 |
| buffer_core | 122 | 0.504 | 0.024 | 0.033 | 0 | 0.279 | 9 |

**Panel C. Linearity assessment**

| **Model** | **Continuous predictor** | **Linear AIC** | **Spline AIC** | **ΔAIC (spline − linear)** |
| --- | --- | --- | --- | --- |
| market_core | Exact-overlap FDCI, per 0.10 | 188.71 | 191.13 | 2.42 |
| market_core | Farm size, ha | 188.71 | 191.71 | 2.99 |
| food_insecurity_core | Exact-overlap FDCI, per 0.10 | 223.68 | 227.63 | 3.96 |
| food_insecurity_core | MFDI, per 0.10 | 223.68 | 226.17 | 2.50 |
| food_insecurity_core | Farm size, ha | 223.68 | 226.88 | 3.21 |
| vulnerability_core | Exact-overlap FDCI, per 0.10 | 216.99 | 218.43 | 1.44 |
| vulnerability_core | Farm size, ha | 216.99 | 219.28 | 2.28 |
| buffer_core | Exact-overlap FDCI, per 0.10 | 215.93 | 217.47 | 1.53 |
| buffer_core | MFDI, per 0.10 | 215.93 | 217.53 | 1.60 |
| buffer_core | Farm size, ha | 215.93 | 219.24 | 3.31 |

**Note.** VIF values near 1 indicate little multicollinearity. Natural-spline specifications were not retained when they did not improve AIC sufficiently to justify the additional model complexity. In modified Poisson models for binary outcomes, Pearson dispersion ratios were treated as diagnostics rather than strict distributional assumptions because inference used robust standard errors.

**Supplementary Table S11.** Influence and farm-size sensitivity analyses for the market-dependence model

**Panel A. Observations exceeding the Cook-distance threshold in the market-dependence model**

| **Record ID** | **Cook distance** | **4/n threshold** | **Leverage** | **DFBETA: FDCI** | **DFBETA: HPDS** |
| --- | --- | --- | --- | --- | --- |
| 90 | 0.481 | 0.032 | 0.872 | -0.027 | -0.002 |
| 2 | 0.035 | 0.032 | 0.168 | 0.490 | 0.336 |

**Panel B. Binary high-market-dependence model after exclusion of influential observations**

| **Scenario** | **Exposure** | **n** | **aPR** | **95% CI** | **p value** |
| --- | --- | --- | --- | --- | --- |
| Full sample | Exact-overlap FDCI, per 0.10 | 124 | 0.933 | 0.874–0.996 | 0.037 |
| Full sample | HPDS, per group | 124 | 0.708 | 0.603–0.832 | <0.001 |
| Exclude household 90 | Exact-overlap FDCI, per 0.10 | 123 | 0.934 | 0.874–0.998 | 0.043 |
| Exclude household 90 | HPDS, per group | 123 | 0.709 | 0.602–0.834 | <0.001 |
| Exclude household 2 | Exact-overlap FDCI, per 0.10 | 123 | 0.921 | 0.866–0.980 | 0.010 |
| Exclude household 2 | HPDS, per group | 123 | 0.694 | 0.591–0.814 | <0.001 |
| Exclude all Cook > 4/n | Exact-overlap FDCI, per 0.10 | 122 | 0.923 | 0.866–0.983 | 0.012 |
| Exclude all Cook > 4/n | HPDS, per group | 122 | 0.694 | 0.590–0.817 | <0.001 |

**Panel C. Continuous-MFDI model after exclusion of influential observations**

| **Scenario** | **Exposure** | **n** | **Coefficient** | **95% CI** | **p value** |
| --- | --- | --- | --- | --- | --- |
| Full sample | Exact-overlap FDCI, per 0.10 | 124 | -0.096 | -0.185–-0.007 | 0.035 |
| Full sample | HPDS, per group | 124 | -0.196 | -0.317–-0.074 | 0.002 |
| Exclude household 90 | Exact-overlap FDCI, per 0.10 | 123 | -0.096 | -0.186–-0.006 | 0.037 |
| Exclude household 90 | HPDS, per group | 123 | -0.195 | -0.317–-0.073 | 0.002 |
| Exclude household 2 | Exact-overlap FDCI, per 0.10 | 123 | -0.111 | -0.202–-0.021 | 0.016 |
| Exclude household 2 | HPDS, per group | 123 | -0.214 | -0.338–-0.089 | <0.001 |
| Exclude all Cook > 4/n | Exact-overlap FDCI, per 0.10 | 122 | -0.111 | -0.202–-0.020 | 0.017 |
| Exclude all Cook > 4/n | HPDS, per group | 122 | -0.213 | -0.338–-0.088 | <0.001 |

**Panel D. Sensitivity to the extreme farm-size observation and functional form**

| **Farm-size specification** | **Predictor** | **n** | **aPR** | **95% CI** | **p value** |
| --- | --- | --- | --- | --- | --- |
| original | Exact-overlap FDCI, per 0.10 | 124 | 0.933 | 0.874–0.996 | 0.037 |
| original | HPDS, per group | 124 | 0.708 | 0.603–0.832 | <0.001 |
| original | Farm size, ha | 124 | 1.001 | 0.985–1.018 | 0.879 |
| excluding_extreme | Exact-overlap FDCI, per 0.10 | 123 | 0.934 | 0.874–0.998 | 0.043 |
| excluding_extreme | HPDS, per group | 123 | 0.709 | 0.602–0.834 | <0.001 |
| winsorised | Exact-overlap FDCI, per 0.10 | 124 | 0.932 | 0.873–0.994 | 0.034 |
| winsorised | HPDS, per group | 124 | 0.707 | 0.601–0.831 | <0.001 |
| log_transformed | Exact-overlap FDCI, per 0.10 | 124 | 0.932 | 0.873–0.994 | 0.033 |
| log_transformed | HPDS, per group | 124 | 0.706 | 0.601–0.828 | <0.001 |

**Note.** The full-sample specification remained the primary analysis because no data error or eligibility violation was identified. Exclusion, winsorization, and logarithmic transformation were diagnostic sensitivity analyses.

**Supplementary Table S12.** Missingness and reliability evaluation

**Panel A. Missingness in variables used in the main models**

| **Variable** | **Missing, n** | **Missing, %** | **Observed unique values** |
| --- | --- | --- | --- |
| agricultural credit | 6 | 4.5 | 2 |
| high market dependence 75 | 4 | 3.0 | 2 |
| high market dependent vulnerability | 4 | 3.0 | 2 |
| low buffer resilience | 4 | 3.0 | 2 |
| mfdi | 4 | 3.0 | 16 |
| farm size ha | 3 | 2.3 | 22 |
| high food insecurity | 2 | 1.5 | 2 |
| fdci overlap | 2 | 1.5 | 15 |
| household size | 2 | 1.5 | 10 |
| agroecological transition index | 2 | 1.5 | 15 |

**Panel B. Reliability and internal-consistency summary**

| **Scale/index** | **Items** | **Measurement model** | **Raw α** | **Standardized α** | **Average inter-item r** | **Interpretation** |
| --- | --- | --- | --- | --- | --- | --- |
| Food insecurity scale | 5 | Reflective | 0.770 | 0.760 | 0.388 | Internal consistency is relevant for this scale |
| Agroecological transition index | 9 | Formative | 0.464 | 0.472 | 0.090 | Descriptive only: the index is treated as formative |
| Financial resilience index | 10 | Formative | 0.580 | 0.594 | 0.127 | Descriptive only: the index is treated as formative |

**Panel C. Food-insecurity scale item statistics**

| **Item** | **n** | **Corrected item-total correlation** | **Mean** | **SD** |
| --- | --- | --- | --- | --- |
| riesgo_actividad_insuficiente | 131 | 0.343 | 0.305 | 0.380 |
| riesgo_no_tres_comidas | 131 | 0.353 | 0.095 | 0.216 |
| riesgo_sin_comida | 131 | 0.658 | 0.324 | 0.429 |
| riesgo_sin_dinero | 129 | 0.771 | 0.430 | 0.441 |
| riesgo_cosecha_insuficiente | 131 | 0.624 | 0.511 | 0.423 |

**Note.** Multiple imputation was not performed because no selected model variable exceeded 5% missingness. Cronbach’s alpha was interpreted for the reflective food-insecurity scale. Alpha estimates for the agroecological-transition and financial-resilience indices are descriptive only because these indices were specified as formative. A polychoric ordinal-alpha estimate was not retained because it was inadmissible and numerically unstable.

**Supplementary Table S13.** Evaluation of the formative vulnerability and buffer-resilience indices

**Panel A. Correlation of leave-one-component-out and alternative-weighting indices with the primary index**

| **Primary formative index** | **Alternative specification** | **Spearman correlation with primary index** |
| --- | --- | --- |
| HMDFVI | Without MFDI | 0.816 |
| HMDFVI | Without food insecurity | 0.804 |
| HMDFVI | Without financial resilience | 0.894 |
| HMDFVI | Market-weighted | 0.943 |
| HMDFVI | Financial-weighted | 0.948 |
| Buffer resilience | Without food security | 0.815 |
| Buffer resilience | Without agroecology | 0.876 |
| Buffer resilience | Without financial resilience | 0.924 |

**Panel B. Exact-overlap FDCI association across alternative continuous HMDFVI specifications**

| **HMDFVI specification** | **n** | **FDCI coefficient per 0.10 increase** | **95% CI** | **p value** |
| --- | --- | --- | --- | --- |
| hmdfvi | 124 | -0.039 | -0.082–0.004 | 0.077 |
| hmdfvi_without_mfdi | 126 | -0.008 | -0.055–0.039 | 0.733 |
| hmdfvi_without_food_insecurity | 124 | -0.056 | -0.103–-0.009 | 0.019 |
| hmdfvi_without_financial | 124 | -0.046 | -0.106–0.014 | 0.131 |
| hmdfvi_market_weighted | 124 | -0.051 | -0.099–-0.003 | 0.038 |
| hmdfvi_financial_weighted | 124 | -0.036 | -0.075–0.004 | 0.080 |

**Note.** The HMDFVI and Buffer Resilience Index were treated as formative composites. Leave-one-component-out and alternative-weighting analyses assessed dependence on particular construction choices; they were not psychometric validation analyses. HMDFVI, Household Market-Dependent Food Vulnerability Index.

**Supplementary Table S14. Item-level construction, scoring, missing-data rules, and interpretation of the study indicators**

**Panel A. Components and scoring of the study indicators.**

| **Index** | **Component or source variable** | **Operational definition** | **Coding and direction** | **Item range** |
| --- | --- | --- | --- | --- |
| **Food Insecurity Index** | Insufficient agricultural or livestock activity (riesgo_actividad_insuficiente) | Respondent disagreed that the household’s agricultural or livestock activity was sufficient to cover the family’s basic needs. | Reverse-coded protective statement: agree = 0; neutral = 0.5; disagree = 1. | 0–1 |
|  | Inability to provide three daily meals (riesgo_no_tres_comidas) | Respondent disagreed that the household was consistently able to provide three meals per day. | Reverse-coded protective statement: agree = 0; neutral = 0.5; disagree = 1. | 0–1 |
|  | Household without food (riesgo_sin_comida) | At least one day during the previous month when the household had no food available. | Adverse statement: disagree = 0; neutral = 0.5; agree = 1. | 0–1 |
|  | Household without money for food (riesgo_sin_dinero) | At least one day during the previous month when the household lacked money to purchase food. | Adverse statement: disagree = 0; neutral = 0.5; agree = 1. | 0–1 |
|  | Insufficient harvest income (riesgo_cosecha_insuficiente) | Harvest returns were insufficient to generate the income required to meet the household’s basic needs. | Adverse statement: disagree = 0; neutral = 0.5; agree = 1. | 0–1 |
| **Agroecological Transition Index** | Environmentally oriented cultivation change (cambio_cultivo_ambiental) | Changes in cultivation practices intended to improve yields or make production more environmentally sustainable. | No = 0; yes = 1. | 0–1 |
|  | Good Agricultural Practices (bpa) | Implementation of Good Agricultural Practices in household production. | No = 0; yes = 1. | 0–1 |
|  | Organic fertilization products (productos_organicos) | Use of organic products for crop fertilization. | No = 0; yes = 1. | 0–1 |
|  | Environmentally appropriate waste disposal (desechos_amigables) | Use of environmentally appropriate methods for disposing of agricultural waste. | No = 0; yes = 1. | 0–1 |
|  | Reduced chemical use (reduccion_quimicos) | Reduction in the use of chemical inputs in crop production. | No = 0; yes = 1. | 0–1 |
|  | Elimination of crop-residue burning (eliminacion_quema) | Elimination of crop or crop-residue burning at the end of the production cycle. | No = 0; yes = 1. | 0–1 |
|  | Soil-conservation practices (conservacion_suelo) | Use of practices intended to conserve or protect agricultural soil. | No = 0; yes = 1. | 0–1 |
|  | Agroecological inputs or practices (aplica_agroecologicos) | Use of agroecological practices or inputs, including green manure, composting, and incorporation of organic matter. | No = 0; yes = 1. | 0–1 |
|  | Training in Good Agricultural Practices (capacitacion_bpa) | Receipt of training in Good Agricultural Practices for household production activities. | No = 0; yes = 1. | 0–1 |
| **Financial Resilience Index** | Current savings (posee_ahorros) | Household currently possessed financial savings. | No = 0; yes = 1. | 0–1 |
|  | Savings sufficient to withstand an income shock (ahorros_suficientes) | Respondent considered the household’s savings sufficient to cope with a negative income shock. | No = 0; yes = 1; assessed among households reporting savings. | 0–1 |
|  | Access to agricultural credit (credito_agricola) | Household had been granted agricultural credit by a formal financial institution during the previous two years. | No = 0; yes = 1. | 0–1 |
|  | Agricultural insurance (seguro_agricola) | Household had agricultural insurance. | No = 0; yes = 1. | 0–1 |
|  | Production sales contracts (contratos_venta) | Household used contracts for the sale of agricultural or livestock production. | No = 0; yes = 1. | 0–1 |
|  | Fair price negotiation (precio_justo) | Household was able to discuss and agree on a fair price for its most recent harvest. | No = 0; yes = 1. | 0–1 |
|  | Technical assistance (asistencia_tecnica) | Household received agricultural extension or technical-assistance services during the previous year. | No = 0; yes = 1. | 0–1 |
|  | Production cost records (registro_costos) | Household maintained records of production costs, profits, or losses. | No = 0; yes = 1. | 0–1 |
|  | Knowledge of production profitability (conoce_ganancia) | Household knew whether each production cycle generated a profit or loss. | No = 0; yes = 1. | 0–1 |
|  | Other household income sources (otras_fuentes_ingreso) | Household had sources of income outside the farm. | No = 0; yes = 1. | 0–1 |

**Panel B. Indicator construction, weighting, missing-data rules, and interpretation.**

| **Indicator** | **Formula and weighting** | **Missing-data rule** | **Range** | **Practical interpretation** |
| --- | --- | --- | --- | --- |
| Food Insecurity Index | Equal-weight mean of five recoded items | Mean of available items; missing when all items were unavailable | 0–1 | Higher values indicate greater food insecurity |
| Agroecological Transition Index | Equal-weight mean of nine binary components | Mean of available components | 0–1 | Higher values indicate greater agroecological transition |
| Financial Resilience Index | Equal-weight mean of ten binary components | Mean of available components | 0–1 | Higher values indicate stronger financial and institutional buffers |
| HMDFVI | ([MFDI + FI + (1-FRI)]/3) | All three components required | 0–1 | Higher values indicate greater market-dependent vulnerability |
| Food System Buffer Resilience Index | ([(1-FI)+ATI+FRI]/3) | All three components required | 0–1; presented ×100 | Higher values indicate stronger buffering capacity |
| FDCI | Exact production–self-consumption overlap divided by HPDS | Missing when HPDS = 0 | 0–1 | Higher values indicate that a larger share of production is self-consumed |

**Note.** Primary indicators were used in the main analyses. Sensitivity indicators were used only to evaluate robustness to alternative operational definitions. Locally constructed indices do not have externally validated population cutoffs.

**Supplementary Figure S1. Spearman correlation structure of primary food-system indicators**

**Note.** Cells show Spearman rank correlations among the primary indicators. Correlations are descriptive and do not imply causal relationships.

**Supplementary Figure S2. Distribution of alternative farm-to-diet coupling definitions**

**Note.** Histograms compare the exact-overlap FDCI, Jaccard similarity, legacy truncated ratio, raw count ratio, and Sørensen similarity. The raw ratio may exceed 1 because it does not require food-group-level correspondence.

**Supplementary Figure S3. Legacy count ratio versus exact production–self-consumption overlap**

**Note.** Triangles identify households whose raw self-consumed-to-produced group-count ratio exceeded 1. The exact-overlap FDCI remains bounded from 0 to 1 because its numerator includes only food groups reported as both produced and self-consumed.

**Supplementary Figure S4.** Sensitivity of the market-dependence association to FDCI definition

**Note.** Points and horizontal lines represent adjusted prevalence ratios and 95% confidence intervals per 0.10-unit increase in each coupling definition. The vertical line represents the null value of 1.

**Supplementary Figure S5. Missingness in variables used in the main models**

**Note.** Bars show the percentage of missing observations for variables used in the main models. Variables with no missing data are displayed at 0%.

**Supplementary Figure S6. Cook's distance across core modified Poisson models**

**Note.** Bars show Cook's distance for each analytical observation. Dashed lines represent the model-specific 4/n threshold. Record IDs are analytical identifiers.

**Supplementary Figure S7. Sensitivity of the market-dependence model to influential observations**

**Note.** Points and horizontal lines show adjusted prevalence ratios and 95% confidence intervals for the exact-overlap FDCI and production diversity in the full sample and after exclusion of observations exceeding the Cook-distance threshold. The full-sample model remained primary.
